# Hepatocyte MMP14 mediates liver and inter-organ inflammatory responses to diet-induced liver injury

**DOI:** 10.1093/pnasnexus/pgae357

**Published:** 2024-08-30

**Authors:** Shannon C Kelly, Cassandra B Higgins, Jiameng Sun, Joshua A Adams, Yiming Zhang, Samuel Ballentine, Yong Miao, XiaoXia Cui, Małgorzata Milewska, Ilona Wandzik, Jun Yoshino, Benjamin M Swarts, Shun-ichi Wada, Brian J DeBosch

**Affiliations:** Department of Pediatrics, Washington University School of Medicine, St. Louis, MO 63110, USA; Department of Pediatrics, Washington University School of Medicine, St. Louis, MO 63110, USA; Department of Pediatrics, Washington University School of Medicine, St. Louis, MO 63110, USA; Department of Pediatrics, Washington University School of Medicine, St. Louis, MO 63110, USA; Department of Pediatrics, Washington University School of Medicine, St. Louis, MO 63110, USA; Department of Pathology and Immunology, Washington University School of Medicine, St. Louis, MO 63110, USA; Genome Engineering and Stem Cell Core, McDonnell Genome Institute, Washington University School of Medicine, St. Louis, MO 63110, USA; Genome Engineering and Stem Cell Core, McDonnell Genome Institute, Washington University School of Medicine, St. Louis, MO 63110, USA; Biotechnology Center, Silesian University of Technology, Krzywoustego 8, Gliwice 44-100, Poland; Faculty of Chemistry, Department of Organic Chemistry, Bioorganic Chemistry and Biotechnology, Silesian University of Technology, Krzywoustego 4, 44-100 Gliwice, Poland; Biotechnology Center, Silesian University of Technology, Krzywoustego 8, Gliwice 44-100, Poland; Faculty of Chemistry, Department of Organic Chemistry, Bioorganic Chemistry and Biotechnology, Silesian University of Technology, Krzywoustego 4, 44-100 Gliwice, Poland; Department of Medicine, Keio University School of Medicine, Minato, Tokyo, Japan; Department of Chemistry and Biochemistry, Central Michigan University, Mt. Pleasant, MI 48859, USA; Institute of Microbial Chemistry (BIKAKEN), Kamiosaki, Shinagawa-ku, Tokyo, 141-0021, Japan; Department of Pediatrics, Washington University School of Medicine, St. Louis, MO 63110, USA; Department of Cell Biology & Physiology, Washington University School of Medicine, St. Louis, MO, USA

**Keywords:** MMP14, energy metabolism, insulin resistance, metabolic dysfunction-associated steatotic liver disease, obesity

## Abstract

The matrix metalloproteinase MMP14 is a ubiquitously expressed, membrane-bound, secreted endopeptidase that proteolyzes substrates to regulate development, signaling, and metabolism. However, the spatial and contextual events inciting MMP14 activation and its metabolic sequelae are not fully understood. Here, we introduce an inducible, hepatocyte-specific MMP14-deficient model (MMP14^LKO^ mice) to elucidate cell-intrinsic and systemic MMP14 function. We show that hepatocyte MMP14 mediates diet-induced body weight gain, peripheral adiposity, and impaired glucose homeostasis and drives diet-induced liver triglyceride accumulation and induction of hepatic inflammatory and fibrotic gene expression. Single-nucleus RNA sequencing revealed that hepatocyte MMP14 mediates Kupffer cell and T-cell accumulation and promotes diet-induced hepatocellular subpopulation shifts toward protection against lipid absorption. MMP14 co-immunoprecipitation and proteomic analyses revealed MMP14 substrate binding across both inflammatory and cytokine signaling, as well as metabolic pathways. Strikingly, hepatocyte MMP14 loss-of-function suppressed skeletal muscle and adipose inflammation in vivo, and in a reductionist adipose–hepatocyte co-culture model. Finally, we reveal that trehalose-type glucose transporter inhibitors decrease hepatocyte MMP14 gene expression and nominate these inhibitors as translatable therapeutic metabolic agents. We conclude that hepatocyte MMP14 drives liver and inter-organ inflammatory and metabolic sequelae of obesogenic dietary insult. Modulating MMP14 activation and blockade thus represents a targetable node in the pathogenesis of hepatic inflammation.

**Significance Statement**

Hepatocyte MMP14 drives peripheral insulin resistance, and hepatic and extrahepatic inflammation, including a direct proinflammatory signal to epididymal white adipose tissue during high-fat and high-sucrose exposure. Additionally, Western diet feeding per se drives hepatocyte subpopulations toward incompetence in urea cycle. MMP14 also drives diet-induced subpopulation shifts, whereas hepatocyte MMP14 deletion under dietary duress preserves the predominant hepatocyte subpopulations observed in lean animals. Lastly, we identify trehalase-stable, trehalose-type glucose transporter (GLUT/SLC2A) inhibitors that reduce hepatocyte MMP14 expression.

## Introduction

Matrix metalloproteinases (MMPs) are calcium-dependent zinc-containing endopeptidases that target extracellular components such as collagens, elastins, gelatins, glycoproteins, and proteoglycans (1). In addition, MMPs are recently implicated in much broader regulation, such as metabolic function through cleavage of hormone signaling proteins and through regulation of other MMPs (2). However, the breadth of regulatory functions for this class of proteins is not completely understood.

MMPs act as secreted or membrane-bound enzymes to exert their proteolytic functions. MMP14 (MT1-MMP) is a type I transmembrane MMP implicated in metabolic and extracellular matrix remodeling (2). In addition to intrinsic direct extracellular matrix substrate tropism, MMP14 cleaves and activates proMMP2 to MMP2 to promote collagen turnover (3). Consistent with expanded physiological utility, MMP14 is expressed in metabolically highly active tissues, such as adipose, liver, and skeletal muscle (SKM). Indeed, MMP14-haploinsufficient mice are protected from high-fat diet-induced fat adiposity (4), and germline whole-body MMP14 KO mice exhibit wasting. Recent data in rodent models and humans indicate that it is induced in insulin-sensitive tissues during aging and it mediates insulin receptor-β cleavage to promote insulin resistance and low-density lipoprotein receptor (LDLR) cleavage to accelerate atherosclerosis in apoE-null mice (2, 5, 6).

MMP14 is ubiquitously expressed, including in developmentally critical tissues, such as placenta. Unsurprisingly, germline whole-body MMP14 KO mice develop early-onset osteopenia and soft tissue frailty and die within a few weeks of birth (7). Thus, the developmental impacts of global or tissue-specific MMP14 deletion represent important barriers to fully understand post-developmental MMP14 function. When juxtaposed with data that demonstrate MMP14 proteolyzes distant extracellular targets via its secretion in exosomes (8), the timing and site of initial MMP14 activation remains a critical barrier to understand MMP14 function in health and to target it in disease. We therefore generated inducible hepatocyte-specific MMP14-deficient (MMP14^LKO^ mice) mice to test the hypothesis that hepatocyte MMP14 deletion is sufficient to attenuate diet-induced obesity and its complications. We show that hepatocyte MMP14 drives diet-induced injury, which includes peripheral adiposity, glucose intolerance, hepatic triglyceride (TG) accumulation, and inflammatory and fibrotic gene expression. Protein binding and mass spectrometry uncovered putative MMP14 substrates in metabolic, immune, and cell surface receptor signaling pathways, and this was independently corroborated by liver transcriptomics mirroring protection from inflammatory gene expression in Western diet (WD)-fed MMP14^LKO^ mice. Strikingly, single-nucleus RNA sequencing (snRNAseq) analyses further revealed that MMP14 promotes hepatocyte-specific subpopulations that transcriptionally suppress urea cycle, while promoting liver T-cell accumulation under dietary duress. These changes associated with attenuated basal and diet-induced SKM and adipose inflammatory gene expression and with impaired inflammatory signaling in a hepatocyte–adipose tissue ex vivo co-culture model. Finally, we demonstrate that biochemically stable trehalose-type glucose transporter (GLUT) inhibitors are translational means to suppress hepatocyte MMP14. We thus establish a model in which to examine hepatocyte MMP14 as a node toward systemic inflammation and conclude that hepatocyte MMP14 conveys deleterious hepatocellular and inter-organ signals to promote systemic inflammation under dietary duress.

## Results

**Fig. 1.**
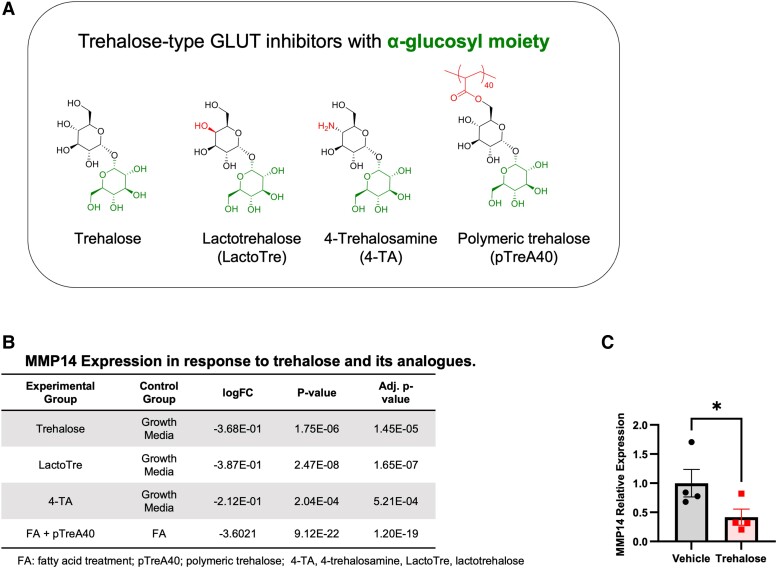
Trehalose analogues inhibit MMP14 expression in vitro and in vivo. A) Haworth projections of trehalose-type GLUT inhibitors with α-glucosyl moieties. The α-glucosyl moiety and functional group that differentiates each carbohydrate are shown. B) Transcriptomic data demonstrating the effect of trehalose (Tre), lactotrehalose (LactoTre), 4-TA, and polymeric trehalose (pTreA40) in isolated primary murine hepatocytes, expressed as log(FC) versus vehicle-treated hepatocytes. C) qRT-PCR data demonstrating hepatic MMP14 expression in response to vehicle or trehalose treatment (3% in water ad libitum) for 0 – 15 days. Data presented as mean ± SEM. *P < 0.05. Statistical analysis: two-tailed t test in (C).

Prior studies demonstrate that GLUT inhibition attenuates hepatic steatosis and dyslipidemia in mice (9–13). We previously identified MMP14 in a transcriptomic screen, because it was differentially suppressed in hepatocytes that were treated with the GLUT inhibitor, trehalose (12–17). The overarching aim of the current study was to assess the extent to which suppressing hepatocyte MMP14 gene expression is sufficient to attenuate diet-induced metabolic disease. The immediate objective in this first set of experiments was to quantify the degree to which hepatic MMP14 expression is pharmacologically modifiable by trehalose-type GLUT inhibitors. One limitation to the clinical use of trehalose, however, is its susceptibility to enzymatic degradation by intestinal brush border trehalases. We therefore tested the hypothesis that stabilized trehalose analogues attenuate MMP14 expression. We quantified the effects of three trehalase-resistant trehalose analogues on hepatic MMP14 expression. This included polymeric trehalose (pTreA40), 4-trehalosamine (4-TA), and lactotrehalose (LactoTre) (Fig. 1A). Transcriptomic analyses in isolated hepatocytes treated with these GLUT inhibitors yielded significantly reduced MMP14 gene expression versus vehicle-treated hepatocytes (Fig. 1B). In addition, polymeric trehalose blocked MMP14 expression [LogFC −3.6, false-discovery rate (FDR) = 1.2e – 19] in bovine serum albumin (BSA)-conjugated fatty acid-treated hepatocytes versus polymer-naïve cultures (Fig. 1B). In vivo, wild-type (WT) mice treated with 3% trehalose in drinking water ad libitum for 15 days similarly suppressed hepatic MMP14 gene expression when compared with vehicle-treated mice (Fig. 1C). The data indicated that hepatic MMP14 expression is pharmacologically modifiable and that trehalose and its analogues reduce MMP14 expression in hepatocytes. Together with data demonstrating that trehalose and trehalose analogues attenuate diet-induced hepatic steatosis, we next sought to define the extent to which hepatocyte MMP14 deletion is sufficient to protect against hepatic lipid accumulation in vivo.

To identify an optimal approach to these experiments, we again noted prior data that demonstrate developmental defects in germline, whole-body MMP14 KO mice (7). This prompted us first to define the extent to which MMP regulation is dynamic during hepatocellular development using an in vitro model of hepatocyte differentiation. To accomplish this, we leveraged an induced pluripotent stem cell (iPSC) model (18) to differentiate human fibroblasts into induced hepatocytes (iHeps). Transcriptomic analysis revealed that MMP14 is among the most significantly up-regulated MMP genes in iHeps versus undifferentiated iPSCs (FDR = 8.2e = 30, Table 1). Robust MMP14 induction during hepatocyte differentiation suggested that MMP14 might be developmentally relevant. In view of these data, we selected a conditional, tissue-specific, post-developmental genetic deletion model to examine MMP14 immunometabolic function in hepatocytes.

**Table 1.** MMP14 is up-regulated during induced hepatocyte differentiation.

Experimental group	Control group	Entrez	Gene name	logFC	*P*-value	FDR
iHep	iPSC	4,312	MMP1	4.70E + 00	1.11E – 05	1.80E – 05
		4,313	MMP2	3.86E + 00	7.20E – 33	1.14E – 30
		4,314	MMP3	5.27E + 00	2.90E – 06	4.93E – 06
		4,316	MMP7	5.22E + 00	4.99E – 05	7.66E – 05
		4,318	MMP9	2.58E + 00	2.76E – 13	8.79E – 13
		4,319	MMP10	4.32E + 00	2.00E – 06	3.44E – 06
		4,320	MMP11	−5.75E – 01	2.20E – 03	2.95E – 03
		4,321	MMP12	9.09E + 00	3.52E – 05	5.46E – 05
		4,323	MMP14	2.46E + 00	6.92E – 32	8.18E – 30
		4,324	MMP15	−9.77E – 01	4.88E – 14	1.67E – 13
		4,325	MMP16	−1.50E + 00	8.90E – 22	8.59E – 21
		4,326	MMP17	−4.67E + 00	1.16E – 22	1.33E – 21
		4,327	MMP19	2.10E – 01	2.87E – 01	3.14E – 01
		9,313	MMP20	3.40E – 01	7.87E – 01	8.06E – 01
		118,856	MMP21	1.14E + 00	2.03E – 01	2.27E – 01
		10,893	MMP24	−1.09E + 00	6.34E – 13	1.96E – 12
		64,386	MMP25	−3.02E + 00	9.38E – 12	2.59E – 11

Shown are transcriptomic data depicting log(FC) relative expression of the MMP genes detected in induced hepatocytes (iHeps) versus induced pluripotent stem cells (iPSCs). The data indicate that MMP14 is among the most highly up-regulated and highly significant (FDR = 8.2E – 30) in iHeps versus iPSCs.

iHep, inducible hepatocytes; iPSC, inducible pluripotent stem cells.

**Fig. 2.**
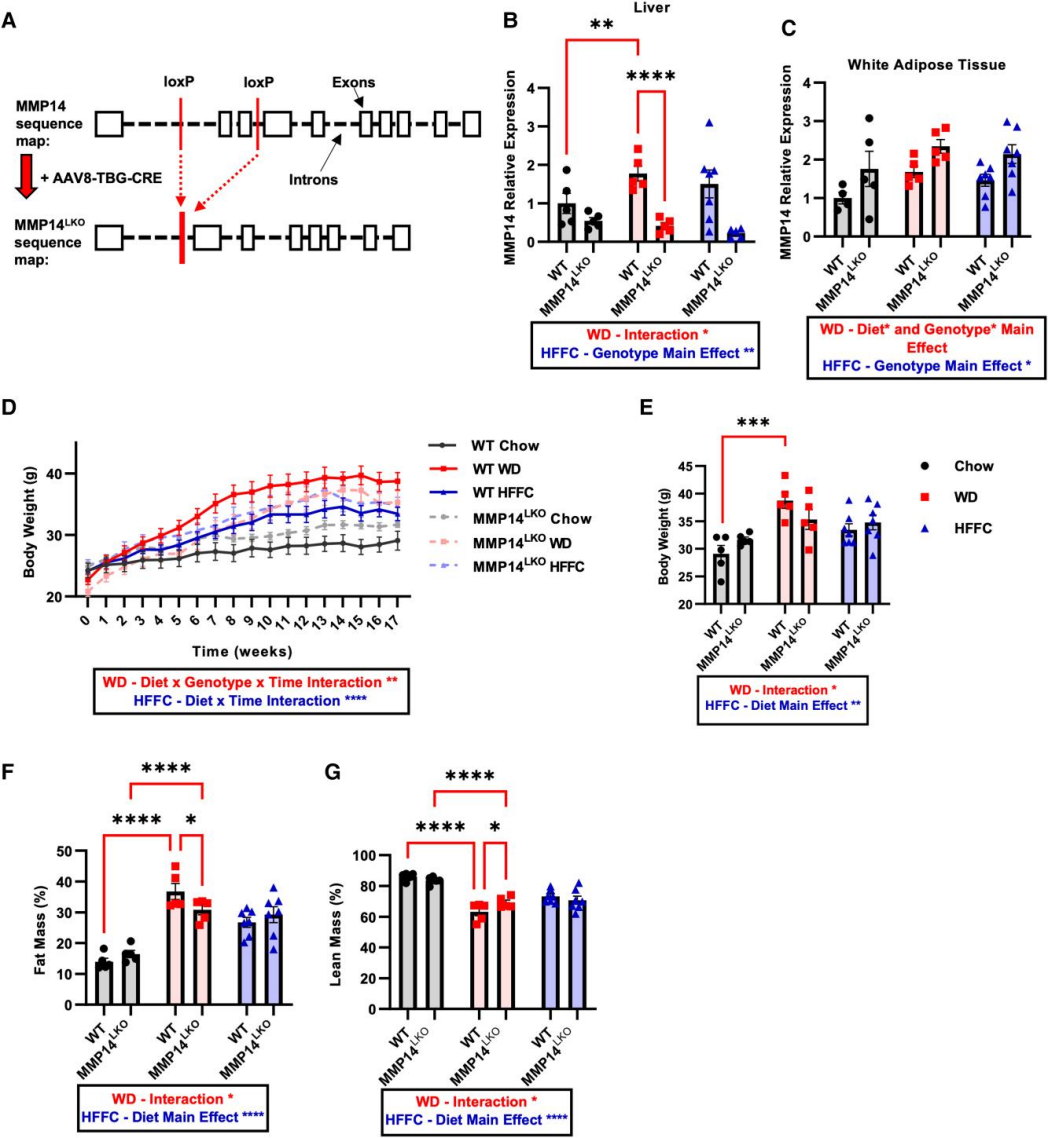
Loss of hepatic MMP14 protects against WD-induced obesity but not HFFC-induced obesity. A) Schematic diagram of exon 2–3 deletion in *MMP14* via AAV8-mediated Cre excision under control of the TBG promoter. B) Liver MMP14 mRNA levels determined by qRT-PCR for WT and MMP14^LKO^ mice fed chow (filled circle), WD (filled square), or HFFC (filled triangle). C) MMP14 mRNA levels in eWAT for WT and MMP14^LKO^ chow, WD, or HFFC. D) Body weight over 17 weeks in WT and MMP14^LKO^ mice fed chow, WD, or HFFC with endpoint body weight quantification (E). F) Fat mass % and G) lean mass percentage determined by EchoMRI for WT and MMP14^LKO^ mice fed chow, WD, or HFFC. *n* = 5 for WT chow, MMP14^LKO^ chow, WT WD, and MMP14^LKO^ WD; *n* = 7 for WT HFFC and MMP14^LKO^ HFFC. Data presented as mean ± SEM. *P < 0.05, **P < 0.01, ***P < 0.001, ****P < 0.0001. Statistical analysis: two-way ANOVA except: D) three-way ANOVA with repeated measures.

We first generated MMP14^fl/fl^ mice by CRISPR–Cas9-mediated *loxP* site insertions flanking exons 2 and 3 (Fig. 2A). We then generated hepatocyte-specific MMP14 knockout mice (MMP14^LKO^) via AAV8-mediated green fluorescent protein (GFP) or Cre overexpression in adult mice, under control of the thyroxine-binding globulin (TBG) promoter. MMP14 mRNA levels were decreased in the liver of MMP14^LKO^ mice (Fig. 2B) without changes in epididymal white adipose tissue (eWAT), indicating hepatocyte-specific deletion (Fig. 2C).

We initiated WT and MMP14^LKO^ mice on standard rodent chow, or one of two distinct, isocaloric diets: WD and high-fat, fructose, and cholesterol (HFFC) diet. We quantified effects of each obesogenic diet to more completely define contextual inputs into MMP14 immunometabolic function. Key macronutrient differences between the diets include differences in fat source (milk fat versus palm oil, lard, and soybean oil), cholesterol content, and sugar content (sucrose versus fructose; Table S1). We observed a significant diet, genotype, and time interaction with regard to body weight trajectory and endpoint body weight in chow- and WD-fed WT and MMP14^LKO^ mice (Fig. 2D and E). In contrast, HFFC-fed MMP14^LKO^ mice were not protected from diet-induced weight gain. In addition, WD-fed MMP14^LKO^ mice exhibited a lower percentage fat mass and higher percentage lean mass after WD exposure, but no protection from fat accumulation was observed in MMP14^LKO^ mice fed with HFFC (Fig. 2F and G).

Lower WD-induced fat accumulation did not correlate with increased adipokine gene expression in eWAT or with changes in substrate oxidation (Figs. S1A–C and S2B) or increased energy expenditure (Fig. S1D–F) during dark or light cycle in WD-fed MMP14^LKO^ mice. Furthermore, WT and MMP14^LKO^ mice had equivalent adiponectin gene expression across chow, WD, and HFFC diet-fed groups, whereas WD-fed MMP14^LKO^ mice had lower leptin gene expression (Fig. S2A and B). Leptin deficiency generally produces accelerated weight gain and impairs glucose homeostasis, and both food intake and total calorie intake were identical between genotypes across all diets (Fig. S2C and D). Therefore, changes in appetite and calorie intake do not likely explain the observed MMP14^LKO^ phenotypes. The data indicate MMP14 protects against peripheral fat accumulation, and this protection is modifiable by diet.

**Fig. 3.**
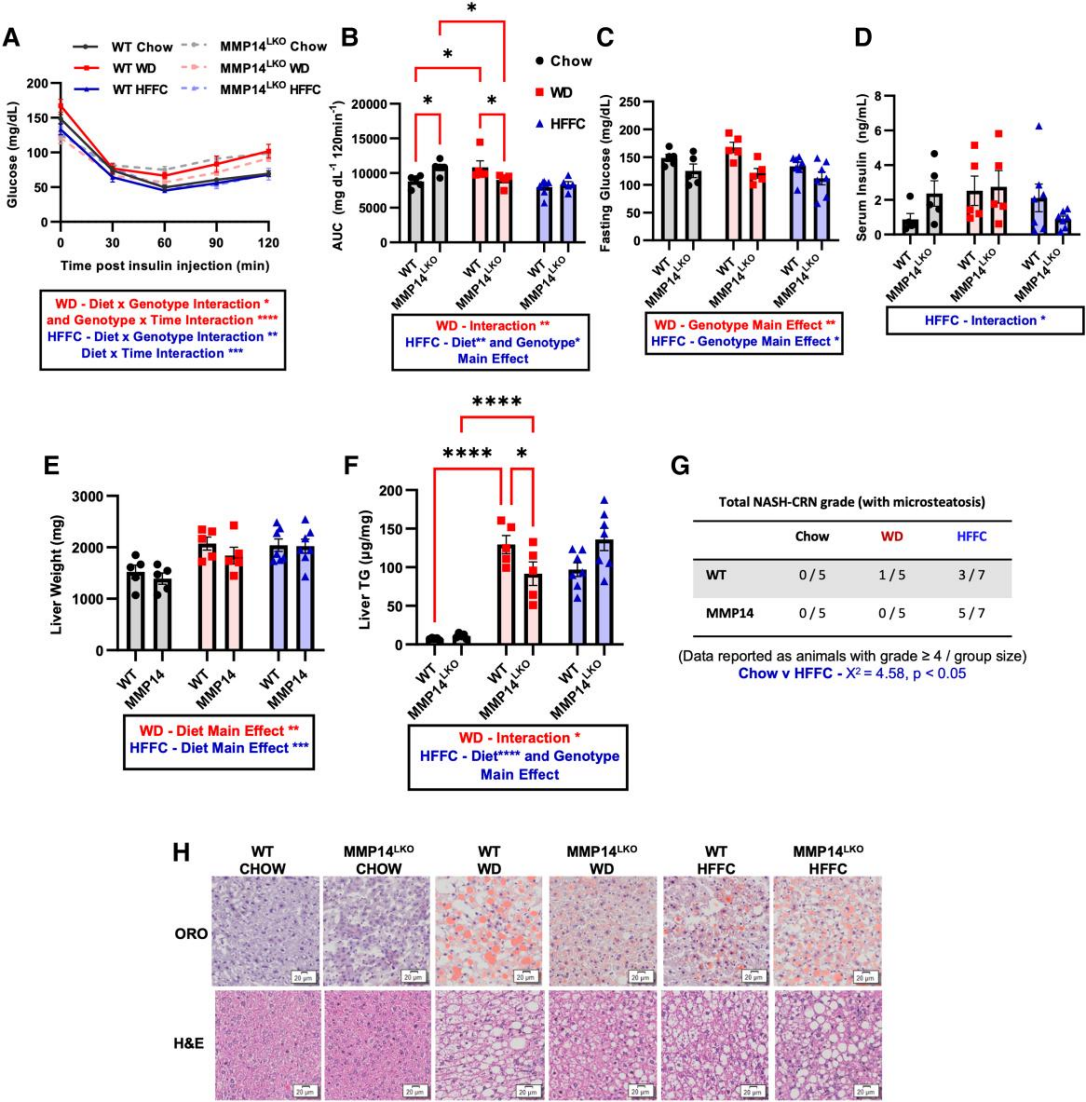
Hepatocyte MMP14 mediates WD-induced insulin resistance and hepatic steatosis. A,B) Insulin tolerance test (ITT) and quantification through area under the curve (AUC) for WT and MMP14^LKO^ mice fed chow (filled circle), WD (filled square), or HFFC (filled triangle). C) Glucose levels after 4-h fasting. D) Serum insulin levels at sacrifice after 17 weeks on diet. E) Liver weights for WT and MMP14^LKO^ mice fed chow, WD, or HFFC. F) Liver TG per milligram of liver weight for WT and MMP14^LKO^ mice fed chow, WD, or HFFC. G) Quantification of inflammatory activity score (NAS score) by blinded histopathologist in H&E-stained liver sections from WT and MMP14^LKO^ mice fed chow, WD, or HFFC (17 weeks). H) Representative images of oil red-O (upper panels), and hematoxylin and eosin (H&E, lower panels) staining in liver sections from WT and MMP14^LKO^ mice fed chow, WD, or HFFC (17 weeks). Data presented as mean ± SEM. *P < 0.05, **P < 0.01, ***P < 0.001, ****P < 0.0001. *n* = 5 for WT chow, MMP14^LKO^ chow, WT WD, and MMP14^LKO^ WD; *n* = 7 for WT HFFC and MMP14^LKO^ HFFC. Statistical analysis: two-way ANOVA except: A) three-way ANOVA with repeated measures and G) χ^2^ analysis.

Insulin tolerance testing (ITT) demonstrated increased insulin sensitivity in WD-fed MMP14^LKO^ mice compared with WD-fed WT mice both by insulin–time curve and by the area under the insulin–time curve (AUC), but not in HFFC-fed animals (Fig. 3A and B). Four-hour fasting data indicated reduced fasting glucose levels in MMP14^LKO^ animals when compared with their WT counterparts on any diet (Fig. 3C). Additionally, we observed a diet–genotype interaction indicating protection from diet-induced hyperinsulinemia in HFFC-fed MMP14^LKO^ mice, when compared with HFFC-fed WT mice (Fig. 3D). No protective effects toward glucose tolerance were observed in response to hepatocyte MMP14 deletion in WD- or HFFC-fed mice (Fig. S3A–D).

We next examined the extent to which MMP14^LKO^ resist WD-induced hepatic steatosis and inflammation. MMP14^LKO^ mice were protected from hepatic TG accumulation in WD-fed, but not in HFFC-fed MMP14^LKO^ liver when compared with equivalent diet-fed WT livers. However, no difference was observed in absolute liver weight and normalized liver weight-to-tibia length ratio (Fig. 3E and F, and Fig. S3E) in WT versus MMP14^LKO^ mice on WD and HFFC diets. We performed blinded histopathologic analysis after hematoxylin and eosin (H&E) and Oil Red-O staining corroborated biochemical analysis of livers from these mice (Fig. 3G and H), and this indicated reduced steatosis in WD-fed liver, but not in HFFC-fed liver versus WT mice. H&E micrographs were scored for lobular inflammation, steatosis, and ballooning (e.g. NAS-CRN grade scoring) by a treatment-blinded hepatopathologist for advanced NASH [e.g. NASH Activity Score (NAS) score ≥ 4]. HFFC-fed animals developed advanced metabolic dysfunction-associated steatohepatitis (MASH) compared to chow-fed mice (*χ*^2^ = 4.57, *P* < 0.05), but this did not differ when comparing WT and MMP14^LKO^ mice (Fig. 3G and H). There were no significant differences in NAS-CRN grade between chow and WD (Fig. 3G and H). Additionally, reductions in hepatic steatosis in WD-fed MMP14^LKO^ mice were not accompanied by protection from diet-induced abnormalities in serum TG, serum cholesterol, serum nonesterified fatty acids, serum low-density lipoprotein cholesterol (LDL-C), serum transaminase elevation, serum albumin (Fig. S4A–F), hepatic cholesterol accumulation, nonesterified fatty acid accumulation (Fig. S5A and B), or changes in hepatic fatty acid oxidation- or de novo lipogenesis-related gene expression in any chow- or diet-induced MMP14^LKO^ group (Fig. S6A–L).

**Fig. 4.**
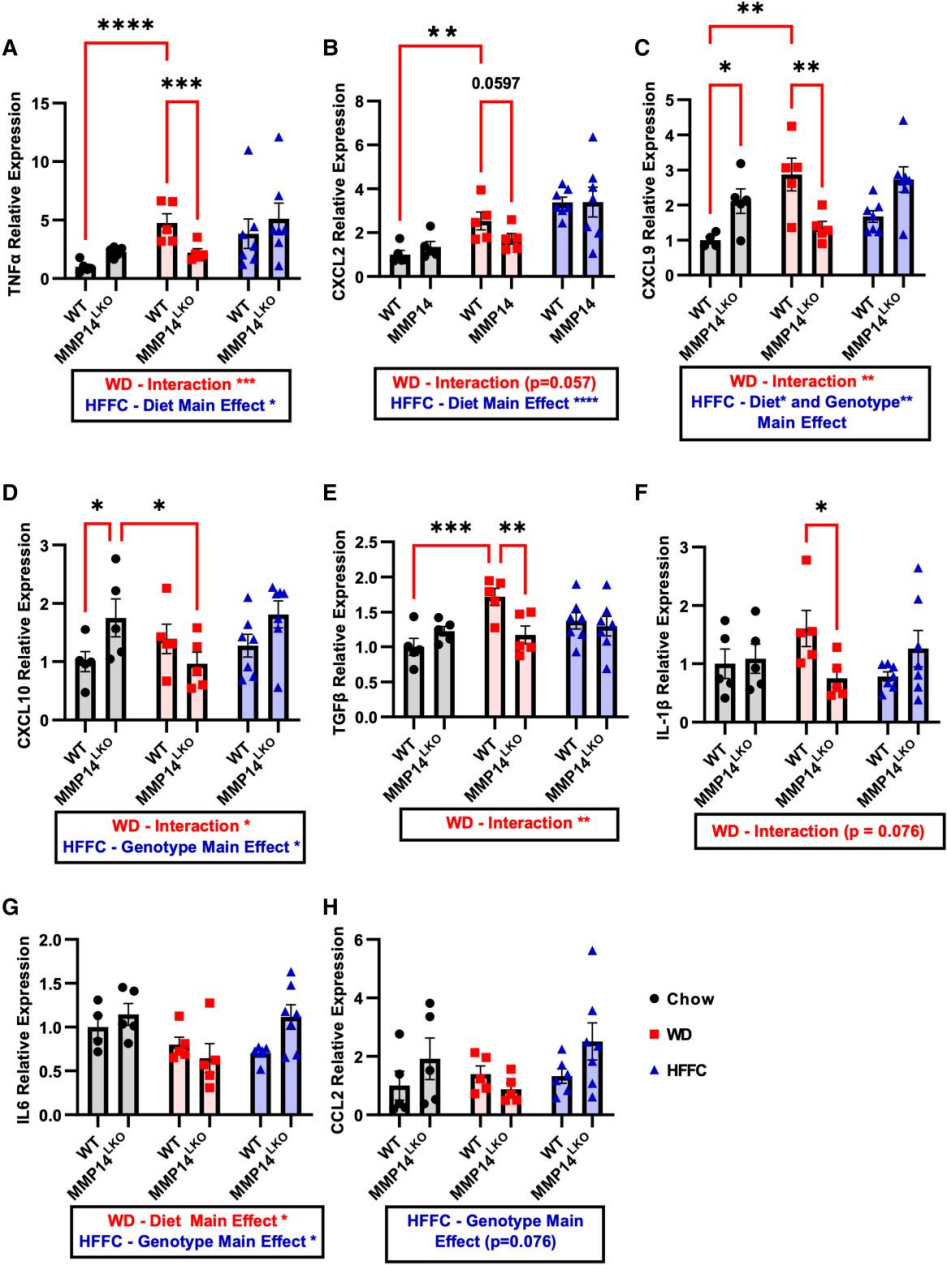
Hepatocyte MMP14 blunts WD-induced, but not HFFC-induced, inflammatory gene expression in liver. Shown are qRT-PCR data demonstrating expression of A) TNFα, B) CXCL2, C) CXCL9, D) CXCL10, E) TGFβ, F) IL-1β, G) IL6, and H) CCL2 mRNA levels in the liver for WT and MMP14^LKO^ mice fed chow (filled circle), WD (filled square), or HFFC (filled triangle). *n* = 5 for WT chow, MMP14^LKO^ chow, WT WD, and MMP14^LKO^ WD; *n* = 7 for WT HFFC and MMP14^LKO^ HFFC. Data presented as mean ± SEM. *P < 0.05, **P < 0.01, ***P < 0.001, ****P < 0.0001.

**Fig. 5.**
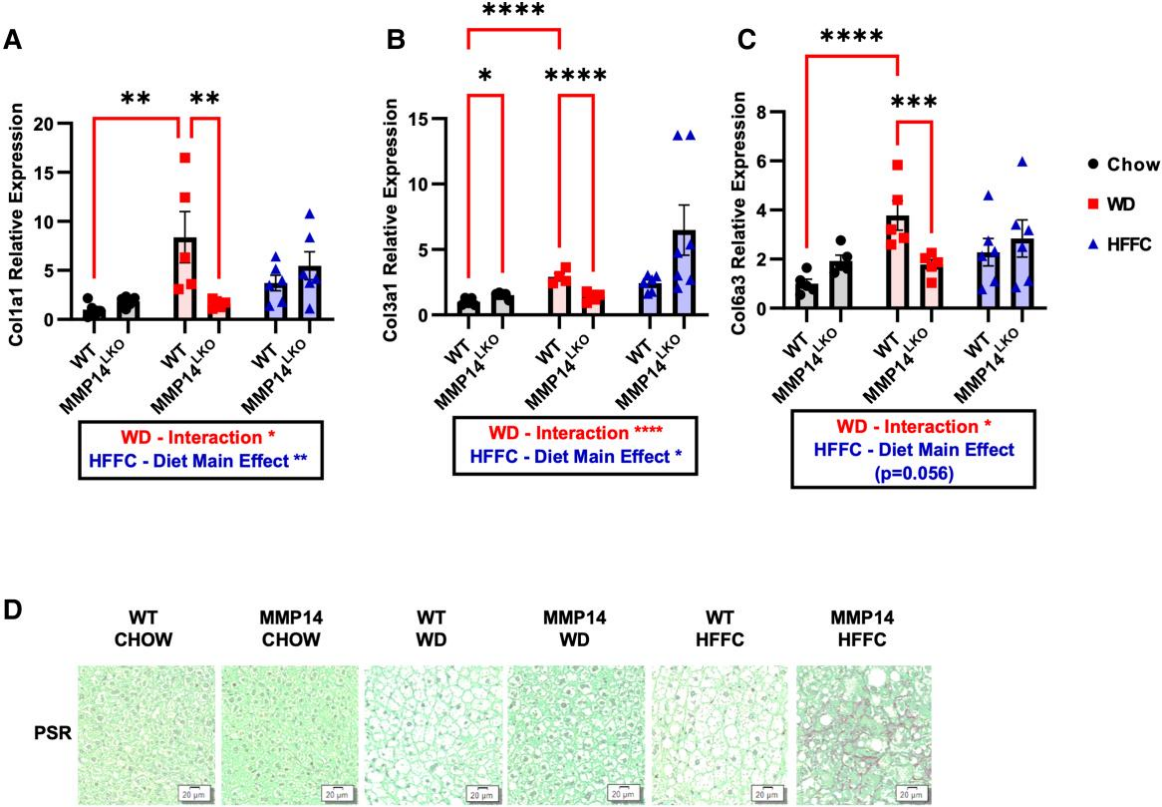
Hepatocyte MMP14 attenuates WD-induced fibrosis but not HFFC-induced fibrosis. Shown are qRT-PCR data demonstrating expression of A) *Collagen 1 (Col1a1)*, B) *Collagen 3 (Col3a1)*, and C) *Collagen 6 (Col6a3)* mRNA levels in liver tissue for WT and MMP14^LKO^ mice fed chow (filled circle), WD (filled square), or HFFC (filled triangle). D) PSR staining in liver sections from WT and MMP14^LKO^ mice fed WD, HFFC, or chow (17 weeks). *n* = 5 for WT chow, MMP14^LKO^ chow, WT WD, and MMP14^LKO^ WD; *n* = 7 for WT HFFC and MMP14^LKO^ HFFC. Data presented as mean ± SEM. *P < 0.05, **P < 0.01, ***P < 0.001, ****P < 0.0001.

Examination of the fibroinflammatory effects of obesity in MMP14^LKO^ mice revealed that both WD and HFFC feeding in mice increased hepatic inflammatory marker gene expression—*TNFα*, *CXCL2*, *CXCL9*, *TGFβ*, and *IL-1β*—in WT mice, and this was attenuated in WD-fed MMP14^LKO^ mice (Fig. 4A–F). Other important inflammatory markers *IL-6* and *CCL2* were not increased by WD feeding (Fig. 4G–H), and thus, no concomitant reduction was observed in their gene expression in MMP14^LKO^ mice. Surprisingly, HFFC diet increased inflammation and this was exacerbated in MMP14 liver-specific knockout (LKO) mice. Similarly, fibrosis-related gene expression—*COL1A1*, *COL3A1*, and *COL6A3* increased in livers of WD-fed mice (Figure 5A–C) and in HFFC-fed mice (Fig. 5A–C). Hepatocyte-specific MMP14 deletion attenuated expression of each of these marker genes in WD-fed MMP14^LKO^ mice relative to WD-fed WT mice (Fig. 5A–C), but not in HFFC-exposed animals (Fig. 5A–C). We corroborated these findings with histologic collagen fiber staining with picrosirius red (PSR). This revealed minimal histologic staining in both WD-fed WT and MMP14^LKO^ groups, lower vacuolization in WD-fed MMP14^LKO^ liver sections, and surprisingly increased histologic PSR staining in MMP14^LKO^ liver sections after HFFC exposure (Fig. 5D).

**Fig. 6.**
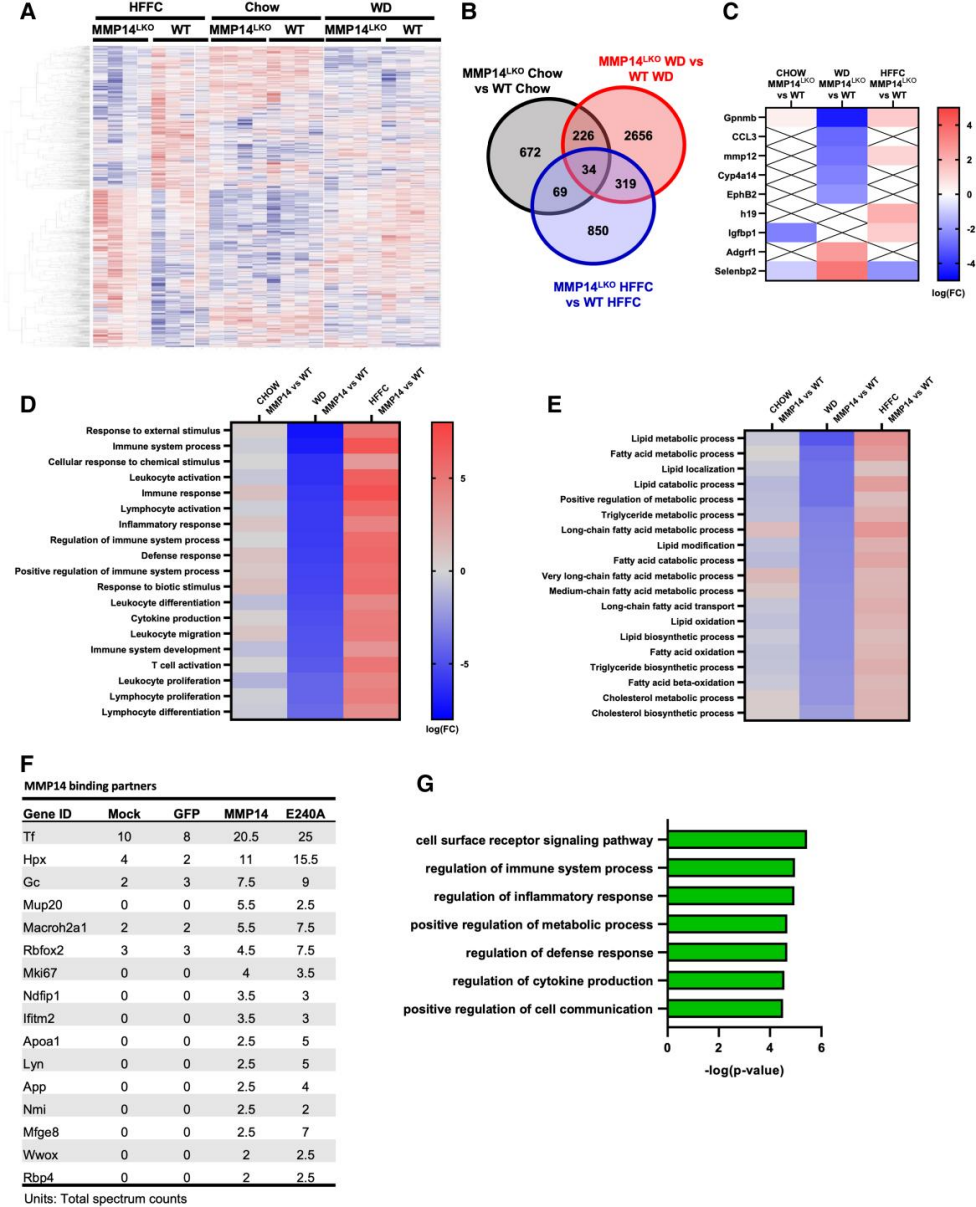
Bulk RNA-seq analysis reveals differential function for hepatocyte MMP14 on inflammatory and metabolic pathway expression in liver during dietary duress. A) Heatmap clustering of differentially expressed genes in liver from MMP14^LKO^ HFFC, WT HFFC, MMP14^LKO^ chow, WT chow, MMP14^LKO^ WD, and WT WD-fed mice. B) Venn diagram representing separation and overlap counts of significantly altered genes (*P* < 0.05) between MMP14^LKO^ and WT for each diet. C) Heatmap of top significantly altered MASLD-associated genes (logFC > 2 and *P* < 0.05 relative to WT dietary control). “X” denotes sequences not significantly altered. D,E) Heatmaps representing top significantly up-regulated and down-regulated inflammatory (D) and metabolic (E) pathways determined by gene ontology analysis. F) Mean MMP14 binding partner hits in GFP immunoprecipitates in hepatocyte cultures that were mock-transfected, GFP-transfected, WT MMP14-transfected, or MMP14^E240A^-transfected. Shown are the mean hits (of two independent biologically distinct immunoprecipitates) in each group. G) Bar graph demonstrating the top up-regulated gene ontology pathways represented when evaluating top protein binding hits identified by proteomics. *n* = 4/group for RNA-seq analyses. Heatmaps presented as log(FC), while bar graph is presented as −log(*P*-value).

We used an unbiased multiomic approach to more comprehensively define how hepatocyte MMP14 modulates the fibroinflammatory response to liver injury. We examined transcriptomic, proteomic, and single-nucleus RNA profiling in livers from WT and MMP14^LKO^ mice after chow, WD, or HFFC feeding. Clustering of differentially expressed genes (DEGs) in livers revealed differentiated groups of DEGs (Fig. 6A). The greatest separation occurred in WD-fed groups, with 2,656 DEGs differentiating WD-fed WT versus MMP14^LKO^ liver, and an additional 226, 319, and 34 DEGs shared when comparing chow-fed, HFFC-fed, and all three groups combined, respectively (Fig. 6B). This included specific reductions in inflammatory genes, *GPNMB*, *CCL3*, *MMP12*, and *EPHB2*, which were up-regulated in humans and animal models of metabolic dysfunction-associated steatotic liver disease (MASLD) or metabolic dysfunction-associated steatohepatitis (MASH) (19–23) (Fig. 6C). In contrast, *GPNMB* and *MMP12* increased in HFFC-fed MMP14^LKO^ versus WT livers (Fig. 6C). *ADGRF1* and *SELENBP1* were down-regulated in humans or animal models of MASLD or MASH (24, 25), and these were up-regulated in MMP14^LKO^ livers versus WT livers fed WD, but not in HFFC-fed MMP14^LKO^ liver. Gene ontology pathway analysis demonstrated attenuation across multiple transcriptomic immune, inflammatory and defense pathway responses in WD-fed MMP14^LKO^ liver, with opposite, significantly up-regulated responses in HFFC-fed MMP14^LKO^ liver, when compared with diet-controlled WT liver (Fig. 6D and E).

Proteomic analysis of putative MMP14 binding partners was performed by expressing GFP-tagged MMP14, a protease activity-deficient point mutant (MMP^E240A^), or no transgene in primary hepatocytes. GFP immunoprecipitation and washout was followed by tryptic digest of bound proteins and mass spectrometry. MMP14 mRNA and protein were verified to be overexpressed (Fig. S7A and B). We then defined putative binding partner peptides as those that were detected in both MMP14- and MMP14^E240A^-expressing hepatocyte immunoprecipitates at a mean value > 2.5-fold greater than in either of the negative control groups (mock-transfected, and GFP-expressing cultures). Top identified hits included proteins that mediate iron homeostasis—such as transferrin and hemopexin ((26) Fig. 6F)—and RBFOX2 (27), which suppresses hepatic cholesterol accumulation and inflammation and is down-regulated in diet-induced obesity. Gene ontology pathway categorization of all proteomic binding partners revealed an MMP14 binding profile toward proteins involved in cell signaling, immune and inflammatory regulation, and cytokine production (Fig. 6G). Together, unbiased bulk multiomic data indicate MMP14 mediates the fibroinflammatory response to diet-induced liver injury, protection from which is diet context-dependent.

**Fig. 7.**
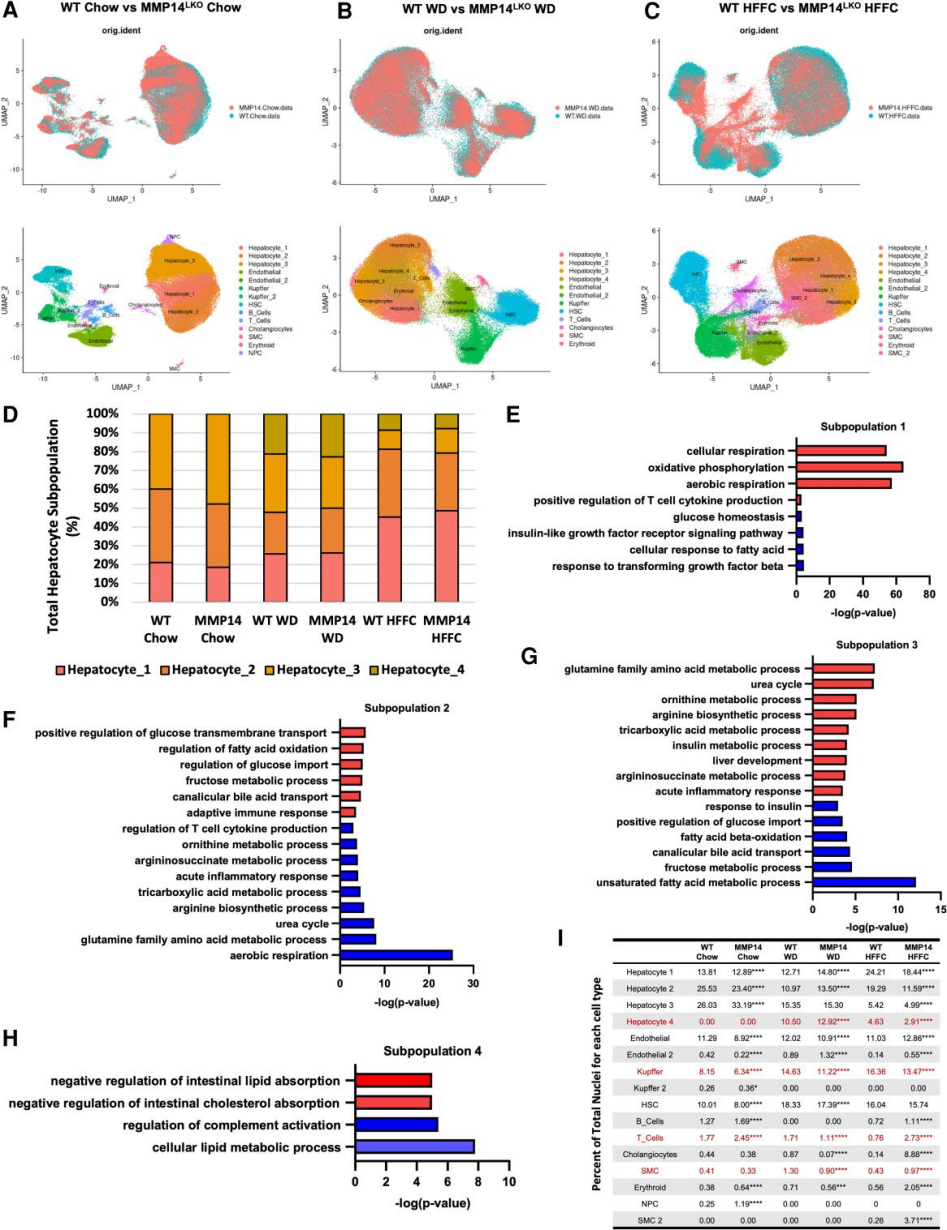
Single-nucleus RNA-seq analysis demonstrated alterations in hepatocyte subtype populations due to the loss of hepatic MMP14 and during dietary duress. A–C) snRNAseq analysis by UMAP clustering of individual cells sequenced from WT and MMP14^LKO^ mice fed chow (A), WD (B), and HFFC (C). Cell population identification in each treatment group is shown in both lower panels. D) Stacked bar graph presenting proportion of each hepatocyte subtype for each experimental group. E–H) Significantly up-regulated (red bars) and down-regulated (blue bars) pathways in hepatocyte subpopulations 1, 2, 3, and 4. I) Percent enrichment of each cell subtype under each dietary and genotype condition. *n* = 3/group for snRNAseq analysis. *P < 0.05, **P < 0.01, ***P < 0.001, ****P < 0.0001 for WT versus MMP14 per diet by Z test for comparing two independent population proportions.

Our data defining a role for MMP14 in physiological hepatocellular differentiation (Table 1) prompted us to test how MMP14 modulates post-developmental cellular responses to liver injury in chow-, WD-, and HFFC-fed livers by single-nucleus RNA-seq (snRNAseq). We observed principal component divergence separating WT and MMP14^LKO^ in each of the identified hepatocyte, Kupffer, stellate, B- and T-cell populations (Fig. 7A–C, A–E and I). We also observed hepatocyte population shifts in livers from mice fed an obesogenic diet, which were not due to alterations in hepatocyte zonality (Fig. S8F). Lean, chow-fed mice of each genotype were enriched for types 1, 2, and 3 hepatocyte populations (Fig. 7D–H). These populations were characterized by gene ontology analysis denoting increased aerobic respiration and oxidative function (types 1 and 2), and urea cycle and nitrogen catabolism (type 3). During WD and HFFC feeding, two major hepatocyte subpopulation shifts occurred. The first was the appearance of lipid-regulatory type 4 hepatocytes, which are characterized by negative regulation of intestinal lipid and cholesterol absorption. The second was a shift toward urea cycle incompetence, by suppressing type 3 hepatocytes (Fig. 7I). Whereas type 3 hepatocyte suppression in WD-fed liver was not significantly attenuated in WD-fed MMP14^LKO^ livers, the appearance of type 4 hepatocytes was significantly increased in WD-fed MMP14^LKO^ livers and was reduced in HFFC-fed MMP14^LKO^ livers (Fig. 7I). These changes correlate with the metabolically protected and unprotected phenotypes in MMP14^LKO^ mice fed WD and HFFC, respectively. In addition, WD-fed MMP14^LKO^ livers had lower T-cell, Kupffer, and smooth muscle cell populations when compared with WD-fed WT livers. Together, snRNAseq data reveal a role for MMP14 in driving both hepatocyte and nonparenchymal cellular landscape evolution in response to dietary liver injury.

**Fig. 8.**
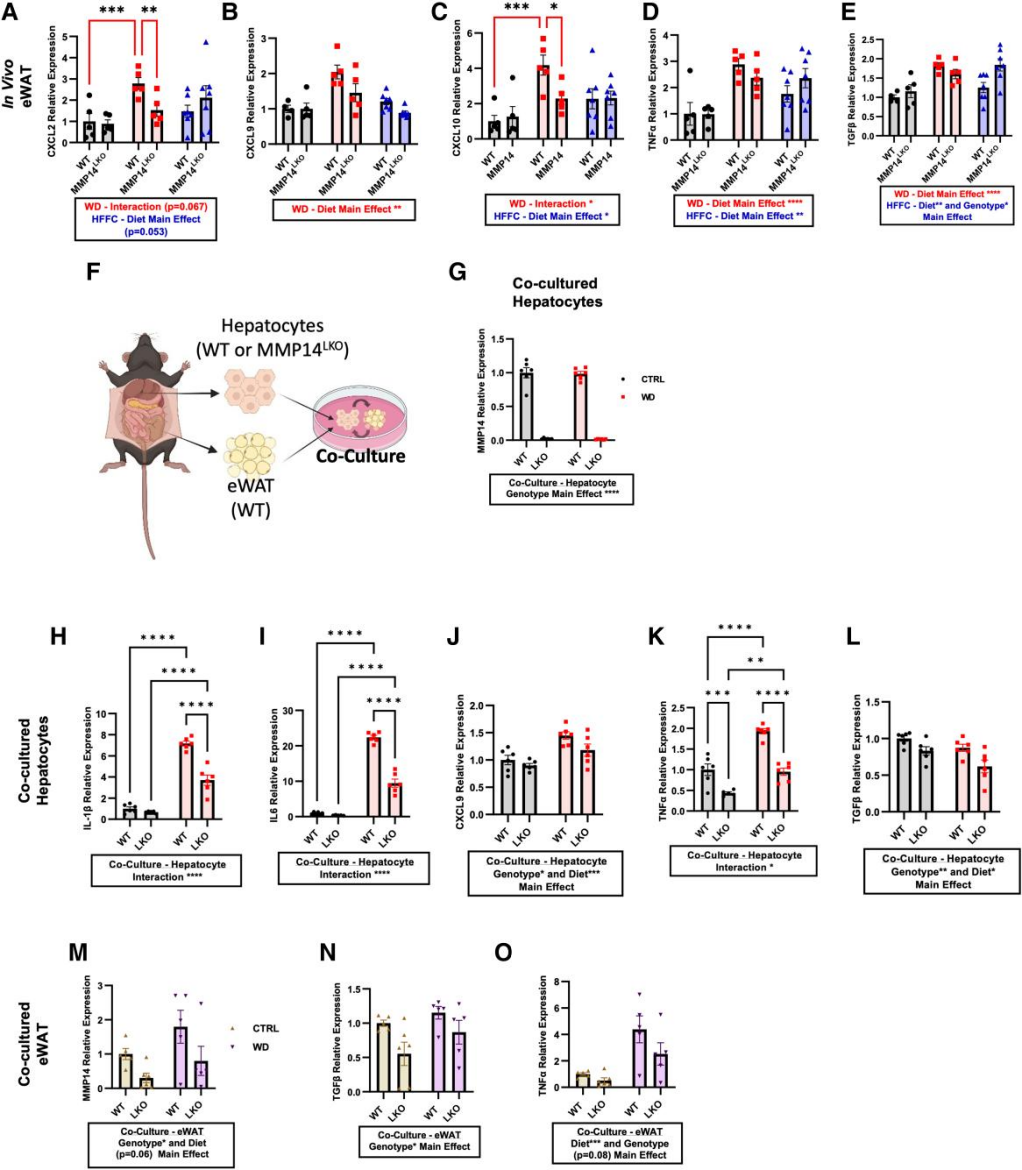
Hepatocyte MMP14 conveys inter-organ inflammatory signaling to white adipose tissue during WD feeding. A) CXCL2, B) CXCL9, C) CXCL10, D) TNFα, and E) TGFβ mRNA expression by qRT-PCR analysis in eWAT from WT and MMP14^LKO^ mice fed chow (filled circle), WD (filled square), or HFFC (filled triangle). *n* = 5 for WT chow, MMP14^LKO^ chow, WT WD, and MMP14^LKO^ WD; *n* = 7 for WT HFFC and MMP14^LKO^ HFFC. F) Schematic diagram of culture of hepatocytes from WT versus MMP14^LKO^ mice together with WT white adipose tissue (WAT). G) MMP14 mRNA levels in WT and MMP14^LKO^ hepatocytes co-cultured with eWAT and treated with or without WD media in vitro. H) IL-1β, I) IL-6, J) CXCL9, K) TNFα, and L) TGF-β mRNA in primary murine hepatocytes from WT mice or MMP14^LKO^ mice co-cultured with WT eWAT and treated with control or WD media in vitro. M) MMP14, N) TGF-β, and O) TNFα mRNA in white adipose tissue co-cultured with primary hepatocytes from WT and MMP14^LKO^ mice treated with control or WD media in vitro. *n* = 6 per group. Data presented as mean ± SEM. *P < 0.05, **P < 0.01, ***P < 0.001, ****P < 0.0001.

snRNAseq data indicated that deleting hepatocyte-specific modulated both hepatocyte and nonhepatocyte responses to dietary injury. These hepatocyte-extrinsic effects observed in MMP14^LKO^ mice prompted broader examination of hepatocyte MMP14 function in adipose and SKM inflammatory responses. WD-induced eWAT inflammatory markers *CXCL2*, *CXCL9*, *CXCL10*, *TNFα*, and *TGFβ* (Fig. 8A–E). Induction of *CXCL2* and *CXCL10* was reduced in eWAT from WD-fed MMP14^LKO^ mice, whereas *CXCL9*, *TNFα*, and *TGFβ* trended lower in WD-fed MMP14^LKO^ eWAT (Fig. 8A–E). In contrast, neither WD nor HFFC induced SKM inflammatory gene expression. Yet, MMP14^LKO^ mice exhibited a genotype-dependent protection and genotype–diet interactions in *CXCL9*, *CXCL10*, and *TGFβ* expression, versus SKM from WT mice (Fig. S9A–E).

We next sought to determine whether hepatocyte MMP14 directly or indirectly drives adipose inflammation. Therefore, we established an ex vivo hepatocyte and adipose co-culture system. We isolated hepatocytes from either WT or MMP14^LKO^ mice and co-cultured them with eWAT from WT mice (Fig. 8F). We treated each co-culture with or without BSA-conjugated fatty acids, sucrose, and cholesterol (i.e. “Western diet” or “WD”) and quantified MMP14 transcript abundance to first confirm *Mmp14* deletion in co-cultured MMP14^LKO^ hepatocytes (Fig. 8G). We then quantified hepatocyte inflammatory marker gene expression in the hepatocyte fraction. This revealed that hepatocyte MMP14 deletion again attenuated WD-induced inflammatory *CXCL9*, *TNFα*, *TGFβ*, *IL-6*, and *IL1-β* expression in co-cultured hepatocytes (Fig. 8H–L).

In eWAT co-cultured with hepatocytes, WD-treatment increased inflammatory marker gene MMP14 and TNFα expression, whereas expression of these genes was significantly attenuated in eWAT co-cultured with MMP14^LKO^ hepatocytes (Fig. 8M and N). We also saw a decrease in TGFβ expression in eWAT co-cultured with MMP14^LKO^ hepatocytes (Fig. 8O). Together, we established in vivo and ex vivo models to define both cell-intrinsic and inter-organ inflammatory signals communicated through hepatocyte MMP14.

## Discussion

Hepatocytes reside at the nexus of portal and systemic circulations to sense and manage inflammatory stimuli—including macronutrients, bacterial by-products, and metabolites—conveyed through the portal circulation. Here, we demonstrate that hepatocyte MMP14 drives a deleterious hepatocyte subpopulation evolution during diet-induced injury and communicates proinflammatory signals to the periphery. Indeed, hepatocyte MMP14 deletion blocked Western diet-induced weight gain and peripheral fat accumulation, insulin intolerance, liver triglyceride accumulation, and hepatic fibrotic and inflammatory gene expression. The data clarify the necessity of hepatocyte MMP14 as a key pathophysiological driver in high-fat, high-carbohydrate contexts that characterize the diet of industrialized nations (28).

In contrast, hepatocyte MMP14 is dispensable toward the deleterious metabolic sequelae rendered by a distinct, isocaloric, MASH-inducing, high-fat, fructose, and cholesterol (HFFC). Although the interaction between cholesterol and MMP14 function is not yet known, prior data show that MMP14 cleaves the LDLR to regulate soluble LDLR and plasma high-density lipoprotein (HDL) and LDL-C (5). We speculate that excess dietary cholesterol may outpace the capacity for retrograde HDL-mediated cholesterol disposal in MMP14^LKO^. Although our model is one of acute (e.g. post-developmental), hepatocyte-specific MMP14 blockade, our data overall extend prior observations to uncover that excessive exogenous cholesterol exposure reverses the antidyslipidemic effects of MMP14 blockade. Therefore, therapeutic application of MMP14 blockade should account for the antecedent dietary exposure. Alternatively, targeting this pathway more proximally in its regulatory pathway—such as through trehalose-type GLUT inhibitors, trehalose, 4-trehalosamine, polymeric trehalose, or lactotrehalose (Fig. 1A–D)—might more broadly leverage MMP14 effects and other molecular effects of blocking hepatocyte carbohydrate metabolism (9, 13–17, 29–44).

We further consider that the expanded metabolic insult rendered by WD feeding, versus the NASH-inducing diet, HFFC (e.g. induction of hepatic steatosis, dyslipidemia, and insulin intolerance), supports an expanded, hepatocyte-extrinsic view of MMP14 metabolic regulation. For example, somewhat surprising is the degree to which MMP14^LKO^ mice suppressed WD-induced inflammatory gene expression in adipose tissue, in basal and WD-exposed SKM, and in vitro co-culture eWAT. In light of the tractability of the liver to genetic and first-pass pharmacological manipulation and in light of the otherwise broader functions of MMP14 in development and other extra-metabolic processes, these data increase the feasibility and impact of precision, post-developmental hepatocyte MMP14 targeting.

MMP14 also mediates changes in the cellular landscape during WD and HFFC feeding across liver cell types. For example, we observed impaired T-cell infiltration in livers of WD-fed MMP14^LKO^ mice when compared with WD-fed WT mice. We also reveal that MMP14 mediates transcriptomic shifts in hepatocyte subpopulations during the evolution of MASLD and MASH. Deleting hepatocyte MMP14 enhanced the appearance of lipid absorption-regulating type 4 populations (Fig. 7I). In contrast, type 3 hepatocytes exhibit increased critical pathway expression, as indicated by nitrogen handling and urea cycle gene expression (e.g. Glul, Slc1a2, Ass1, and Asl). The suppression of urea cycle-competent type 3 hepatocytes across both WD and HFFC corroborates recent work from our group and others recently underscores the importance of nitrogen catabolism to protect against MASLD and MASH, respectively (39, 41, 43, 45–47). Finally, MMP14 deletion in hepatocytes reduced T-cell and Kupffer cell accumulation. We acknowledge these data are associative at present, and thus, future work will, in part, examine the extent to which these subpopulations shifts toward urea cycle incompetence specifically contribute to MASH pathogenesis. Moreover, the intermediary (e.g. transcription factor) targets through which MMP14 exerts this effect are yet to be identified. Nevertheless, we hypothesize that the data offer a framework wherein posttranslational proteolytic modification can drive liver population evolution in the transition from health to disease.

Finally, we previously demonstrated that oral trehalose (14, 15, 17) and lactotrehalose (12, 13, 48) attenuate hepatic injury under dietary duress by blocking glucose transporters in the SLC2A family of facilitative transporters. Polymeric trehalose also blocked inflammatory cell surface signals, such as CD53, during NASH (44). Our new data indicate trehalose and its analogues reduce liver MMP14 expression in vitro and in vivo. Additionally, although a key impact of this work is to demonstrate the role of hepatocyte MMP14 in hepatic steatosis, body weight gain, and inter-organ inflammatory responses, these findings provide one potentially translatable means to reduce hepatocyte MMP14 expression in diet-induced peripheral adiposity, glucose intolerance, liver TG accumulation, and inflammatory and fibrotic gene expression. This translatability is further underscored by recent data, which identified polymorphisms in the *Mmp14* gene that associate with obesity and metabolic disease in human cohorts (4). In sum, we have demonstrated an inter-organ and hepatocyte-intrinsic dual inflammatory and metabolic function for MMP14 that is pharmacologically modifiable, and we provide novel agents with which to achieve MMP14 targeting to potentially abate obesity and its complications.

## Materials and methods

Eight-week-old MMP14^fl/fl^ mice were injected with AAV8-TBG-GFP or AAV8-TBG-CRE via tail vein to generate hepatocyte-specific MMP14 knockout mice (MMP14^LKO^). Two weeks after injection, mice were fed ad libitum: a standard chow diet; Western diet (WD); or a high-fat, fructose, and cholesterol (HFFC) diet for 17 weeks. Two-way ANOVA was utilized to analyze interactions between diet (e.g. chow/WD and chow/HFFC) and genotype. More detailed methods, including primer sequences (Table S2) are given in the Supplementary Methods. All animal protocols were approved by the Washington University School of Medicine Animal Studies Committee.

## Supplementary Material

pgae357_Supplementary_Data

## Data Availability

All data are included in the manuscript and/or supporting information.
