# Supplementary material for: Hepatocyte MMP14 mediates liver and inter-organ inflammatory responses to diet-induced liver injury
Source: PNAS Nexus. 2024 Aug 30;3(9):pgae357. doi: 10.1093/pnasnexus/pgae357 (PMC11393575; doi:10.1093/pnasnexus/pgae357)
Supplement: pgae357_Supplementary_Data [file pgae357_supplementary_data.pdf]

PNAS nexus

Supplementary Information for
Hepatocyte MMP14 mediates liver and inter-organ inflammatory responses to diet-induced liver injury

Shannon C. Kelly[1], Cassandra B. Higgins[1], Jiameng Sun[1], Joshua A. Adams[1], Yiming Zhang[1], Samuel Ballentine[2], Yong Miao[3], XiaoXia Cui[3], Małgorzata Milewska[4,5], Ilona Wandzik[4,5], Jun Yoshino[6], Benjamin M. Swarts[7], Shun-ichi Wada[8], and Brian J. DeBosch[1,9*]

[1]Department of Pediatrics, Washington University School of Medicine, St. Louis, MO 63110
[2]Department of Pathology and Immunology, Washington University School of Medicine, St. Louis, MO 63110
[3]Genome Engineering and Stem Cell Core, McDonnell Genome Institute, Washington University School of Medicine, St. Louis, MO 63110
[4]Biotechnology Center, Silesian University of Technology, Krzywoustego 8, 44-100 Gliwice, Poland
[5]Department of Organic Chemistry, Bioorganic Chemistry and Biotechnology, Faculty of Chemistry, Silesian University of Technology, Krzywoustego 4, 44-100 Gliwice, Poland
[6]Department of Medicine, Keio University School of Medicine, Minato, Tokyo, Japan.
[7]Department of Chemistry and Biochemistry, Central Michigan University, Mt. Pleasant, Michigan
[8]Institute of Microbial Chemistry (BIKAKEN), Kamiosaki, Shinagawa-ku, Tokyo, 141-0021 Japan
[9]Department of Cell Biology & Physiology, Washington University School of Medicine, St. Louis, MO 63110

*Corresponding Author: Brian DeBosch

Email: deboschb@wustl.edu

This PDF file includes:

Supplementary text
Figures S1 to S9
Tables S1 to S2
SI References

Supplementary Information Text

SUPPLEMENTARY METHODS

Animal Studies. All animal protocols were approved by the Washington University School of Medicine Animal Studies Committee. CRISPR reagents for generating MMP14$^{fl/fl}$ mice were designed and validated by the Genome Engineering & Stem Cell Center at the McDonnell Genome Institute (GESC@MGI) at Washington University in St. Louis. Mice were generated at the Transgenic, Knockout and Microinjection Core at Washington University in St. Louis, and genotyped by GESC@MGI through methods previously described (12). The following two gRNA target sites are in introns 1 and 3, respectively: 5'-TTGACTCAATACAACTAACGNGG and 5'-ACAAGAGCGCTGCTCCACCANGG. The two single stranded oligodeoxynucleotides (ssODNs) used as donor templates have the following sequences:

5'-
c*a*ggtcctaagtcagctgctcctgaatcacccccagaccccacacaagagcgctgctccaATAACTTCGTATAATG TATGCTATACGAAGTTATGGATCCccacgggtgcctaagtctgaaggagaagagcatcatatggatccatagat ccatcctaatga*t*c

And

5'-
c*a*ctttctggtgagtcccccagcctagagatgtttttcacatgcagcatccctccccgtATAACTTCGTATAATGTATG CTATACGAAGTTATGGATCCtagttgtattgagtcaaaggtggcctgggctactgtccattcctagagagtggctctaa t*t*c.

Each asterisk represents a phosphorothioate bond. Mice were housed at the Washington University School of Medicine in St. Louis in a 12-h alternating light-dark cycle and temperature-controlled facility on standard cob bedding throughout the experiment. Procedures were performed in accordance with approved guidelines by the Animal Studies Committee (Washington University School of Medicine). Animal studies were performed in accordance with ethical regulations outlined by the Institutional Animal Care and Use Committee (IACUC).

Adeno-associated virus 8 (AAV8) under the thyroid binding globulin (TBG) promoter overexpressing GFP (AAV8-TBG-GFP) or Cre (AAV8-TBG-Cre) were obtained as ready-to-use viral stocks (Vector BioLabs; AAV8-TBF-GFP #VB1743; AAV-TBG-iCRE #VB1724). Eight-week-old male mice were injected with 10^{11} particles of AAV8-TBG-GFP or AAV8-TBG-CRE via tail vein 8-10 days prior to diet treatments as previously described (13-16). At 10 weeks of age, mice were fed ad libitum: a standard chow diet (Lab Diet); Western diet (WD; Inotiv TD.88137); or a high-fat, fructose, cholesterol diet (HFFC; Research Diets D09100310) for 17 weeks. Dietary components are in Supplemental Table 1. Mice had access to free water throughout the experiment.

Hepatocyte isolation, co-culture and treatment. As previously described, primary hepatocytes were isolated from wild-type mice or MMP14$^{fl/fl}$ mice bred with Albumin-cre mice which generated germline, hepatocyte-specific MMP14^{LKO} mice (13, 15, 17, 18). Cells were plated at 5 x 10^5 cells per well in 12-well plates and maintained in regular Dulbecco's modified Eagle's growth media (Sigma; #D5796) with 10% fetal bovine serum. After 24 h, cells were treated with components replicative of WD or control (CTRL) media without dietary components. For WD, cells were treated with 5 mM sucrose, 2.5 μM cholesterol (Sigma; #C4951), 500 μM BSA-conjugated free fatty acids (69.7 μM myristic acid, 190.2 μM palmitic acid, 82.3 μM stearic acid, 137.5 μM oleic acid, and 15.1 μM linoleic acid), and 50 ng/mL LPS (19). In conjunction with dietary treatment, cells were incubated with 25 mg of epididymal WAT rinsed in warm PBS from WT mice. WAT was suspended above the cultured hepatocytes for

a co-culture model (20, 21). Cells and WAT were incubated in dietary components for 24 hours and then lysed in Trizol for downstream analysis. The fatty acid components were obtained from the following: myristic acid (Sigma; #70082), palmitic acid (Sigma, #76119), linoleic acid (Sigma; #62230), stearic (ThermoFisher, #A12244.06), and oleic acid (ThermoFisher, #031997.06). iHep differentiation from human fibroblasts was undertaken based on prior published protocols through the Washington University Genome Engineering and iSPC core facility (22, 23). We synthesized and purified trehalose analogues as previously reported (24-27).

Body Composition analysis. Unanesthetized mice were subject to EchoMRI for body composition analysis using the EchoMRI 3-1 device (Echo medical Systems, Houston, TX) via the Washington University Diabetic Mouse Models Phenotyping Core Facility.

Insulin and glucose tolerance testing. Insulin tolerance tests were conducted by intraperitoneal injections 0.75 IU per kg BW of insulin (Lilly USA, LLC) after mice fasted for 4 h on aspen bedding. Glucose tolerance tests were conducted by intraperitoneal injections of 2 g per kg BW glucose after 6 h fasting on aspen bedding. Fasting blood glucose levels were measured prior to injection with a glucometer (Arkray USA, Inc.) and at 30' intervals post-injection.

Indirect calorimetry. A PhenoMaster System (TSE systems) was utilized to measure metabolic performance and activity by an infrared light = beam frame. Mice were placed in separate chambers of the open-circuit calorimetry. Mice were maintained at room temperature (22-24 °C) and provided food and water ad libitum. Mice were allowed to acclimatize in the chambers for 24 hours. The parameters of indirect calorimetry (respiratory exchange ratio (RER) and heat) were measured for at least 24 h including one light and one dark cycle. Data is presented as average values over the light and dark cycles.

Clinical chemistry and hepatic lipid analysis. Prior to sacrifice, submandibular blood was collected and the serum was separated. Insulin ELISA (Millipore; #EZRMI-13K), triglyceride (ThermoFisher; #TR22421), cholesterol (ThermoFisher; #TR13421), free FA (Wako Diagnostics; #999-34691, #995-34791, #991-34891, and #993-35191), LDL-C (Wako Diagnostics; #999-00504, #993-00404), albumin (Sigma; #MAK124), glucose (Cayman Chemical; #10009582), and ALT (Cayman Chemical; #700260) quantification were performed using commercially available reagents according to the manufacturer's instructions.

Hepatic lipids were extracted from ~100 mg of snap-frozen hepatic tissue homogenized in 2:1 chloroform: methanol. Extracts (0.25%–0.5% of sample) were evaporated overnight prior to biochemical quantification of triglycerides, cholesterol, and FFA using reagents described above, according to manufacturer's directions.

Quantitative real-time RT-PCR (qPCR). Total RNA was prepared by homogenizing snap-frozen livers or cultured hepatocytes in Trizol reagent (Invitrogen; #15596026) according to the manufacturer's protocol. cDNA was prepared using Qiagen Quantitect reverse transcriptase kit (Qiagen; #205310). Real-time qPCR was performed with QuantStudio 3 Real-Time PCR System (Applied Biosystems) using SYBR Green master Mix Reagent (Applied Biosystems) and specific primer pairs. Relative gene expression was calculated by a comparative method using values normalized to the expression of an internal control gene. Primers utilized have been previously published (13, 15, 28) or can be found in Supplemental Table 2.

Histological Analysis. Formalin-fixed paraffin-embedded liver sections were stained by H&E and picrosirius red (PSR) via the Washington University Digestive Diseases Research Core Center. OCT-embedded frozen liver sections were stained by Oil Red O according to standard protocols flowered by microscopic examination. Three liver sections were examined and

evaluated for each animal. For Oil red O staining, ice-cold methanol-fixed frozen sections from mice were stained according to described protocols (19, 29, 30). A treatment-blinded, board-certified GI/Liver pathologist scored each liver section for inflammation, steatosis, and ballooning. The sum of the scores for inflammation, steatosis (with microsteatosis), and ballooning are presented as the total NASH-CRN grade where NASH-CRN grade ≥ 4 is indicative of NASH (31).

RNAseq and Single-nucleus RNAseq analysis (snRNAseq). Liver RNA-seq was performed on snap-frozen liver tissue by the Washington University Genome Technology Access Center as previously described (13, 15, 18, 28). For snRNAseq, liver nuclei were extracted for snRNAseq from snap-frozen liver sections utilizing the nuclei isolation protocol for single cell gene expression (10x Genomics; #PN-1000494) per manufacturer's instructions. Three liver sections from three distinct animals per treatment group were combined for the analysis. A total of ~20,000 nuclei were utilized per treatment group. Library preparation and sequencing were performed by the Washington University Genome Technology Access Center. Libraries from isolated nuclei were prepared through the 10X 3' v3.1 Single Nuclei pipeline (10X Genomics) and sequenced using Illumina technology.

snRNAseq was analyzed in RStudio (R version 4.0.3, Boston, MA). Cell Ranger outputs were first processed with SoupX package(32) before proceeding with the Seurat pipeline (33). Before integrating by diets, datasets were filtered with the parameters nFeature_RNA > 750 & nFeature_RNA < 3000 & percent.mt < 1.5 and percent.hb < 0.1. Clustering was then performed on the first ~30 principal components and visualized with UMAP. All data was combined for hepatocyte subgroup analysis and pathways were analyzed with Gene Ontology Enrichment Analysis.

Immunoprecipitation. Cultured primary hepatocytes from WT mice were treated with 10^7 particles of adenovirus overexpressing GFP (adGFP), MMP14-tagged GFP (adMMP14), or catalytically inactive MMP14 (8) with GFP tag (adE240A) (Vector BioLabs; GFP #1060; MMP14 #ADV-264720; E240A was generated by Vector BioLabs) and incubated for 24 hours. Overexpressing adGFP, adMMP14, and adE240A were lysed with lysis buffer (Cell Signaling, #9803) and protein concentration was determined via BCA Assay kit. 500 µg of protein was incubated with biotin anti-GFP antibody (Abcam, #ab6658) at 4 °C overnight. Samples were then incubated with 20 µL Pierce streptavidin magnetic beads (Thermo Scientific, #88816). After incubation and washing, protein was released from beads with 50 µL 2x Laemmli Buffer and heating at 99 °C for 5 min. 40 µL of sample 4-20% TGX stain-free gels prior to further processing for immunoblotting as described earlier. MMP14 primary antibody (Cell Signaling, #13130) was utilized to verify MMP14 overexpression and Coomassie staining was utilized as loading control.

Proteomics. Immunoprecipitated primary hepatocytes overexpressing adGFP, adMMP14, adE240A, or no adenovirus were prepared for proteomic analysis via mass spectrometry as previously described (18). Peptides were filtered at 1% false discovery rate by searching against a reversed protein sequence database, and a minimum of two peptides were required for protein identification. Protein binding hits were filtered based on binding of both MMP14 and E240A and the binding hit was at least 2.5-fold higher than that observed in the negative controls: control (no adenovirus) and adGFP.

Statistical Analysis. All data were analyzed using GraphPad Prism 9.0. $p < 0.05$ was defined as statistically significant, unless otherwise specified. Data is presented as mean ± SEM. Two-way ANOVA was utilized to analyze interactions between diet (e.g., Chow/WD and Chow/HFFC) and genotype. Significant interactions prompted post hoc analysis with Fisher's LSD test. In cases wherein no significant two-way interaction is detected, only main effects are reported. Specific statistical tests applied are noted in the Figure Legends.

SUPPLEMENTAL FIGURE 1

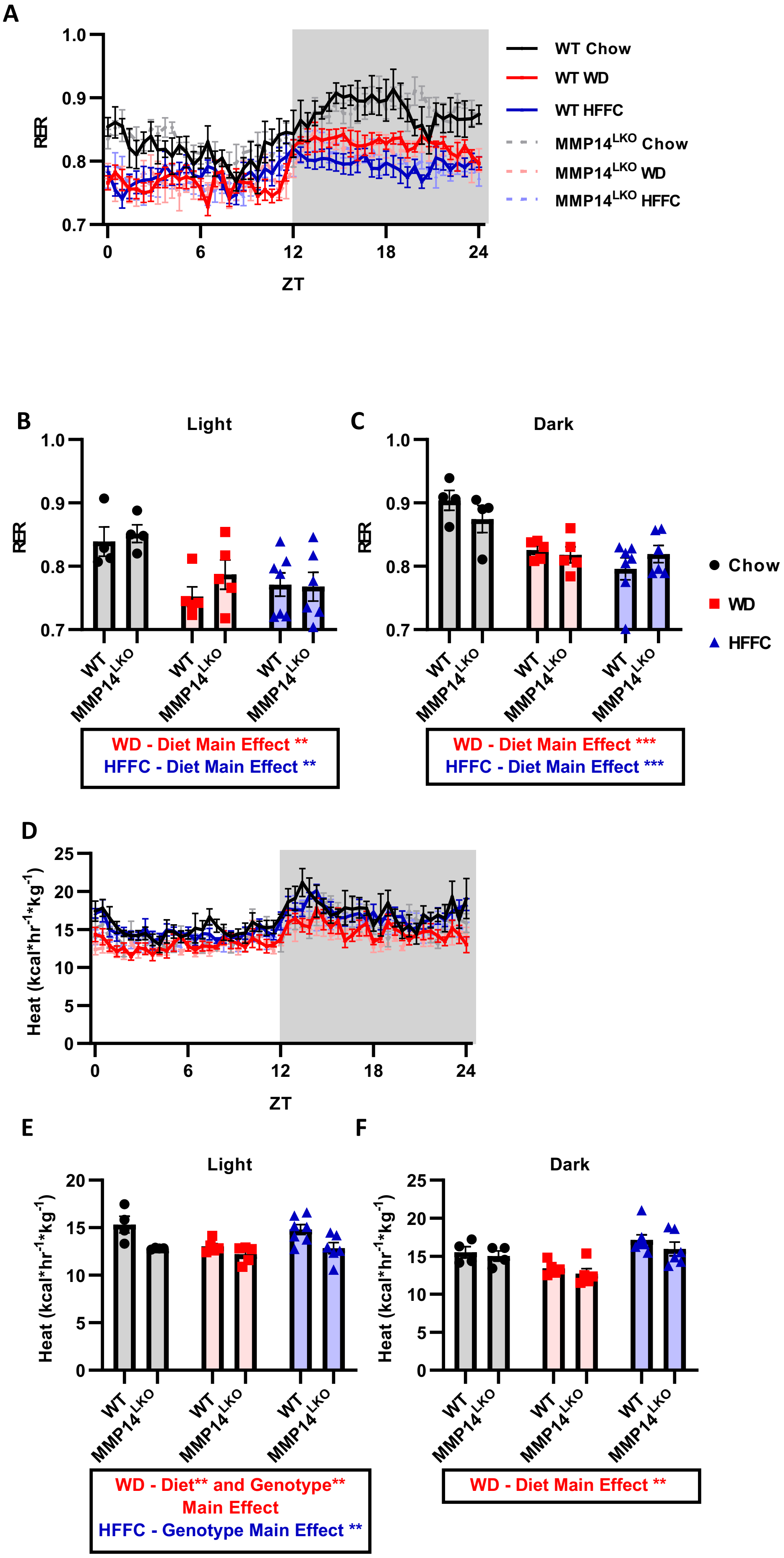

SUPPLEMENTAL FIGURE 2

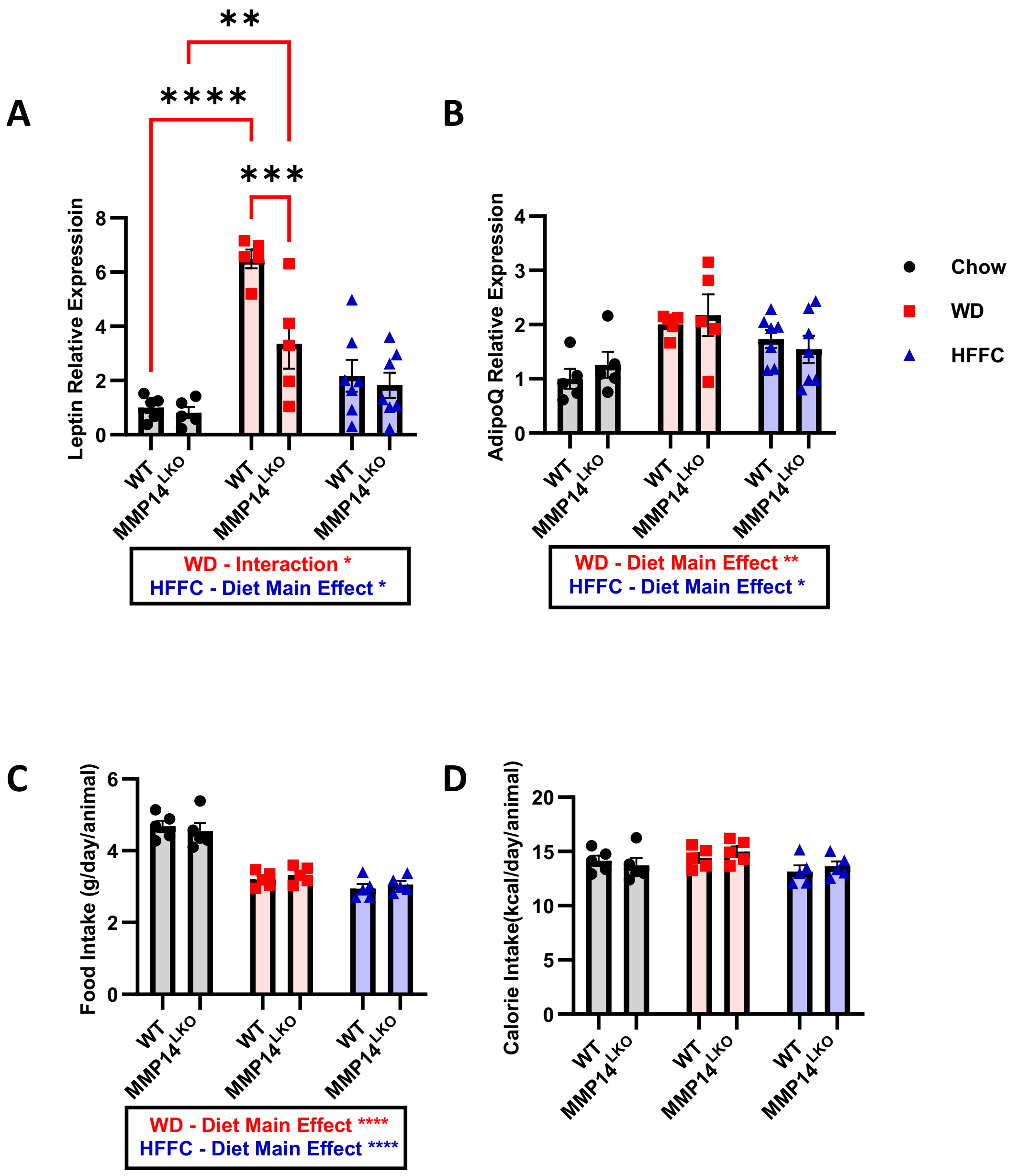

SUPPLEMENTAL FIGURE 3

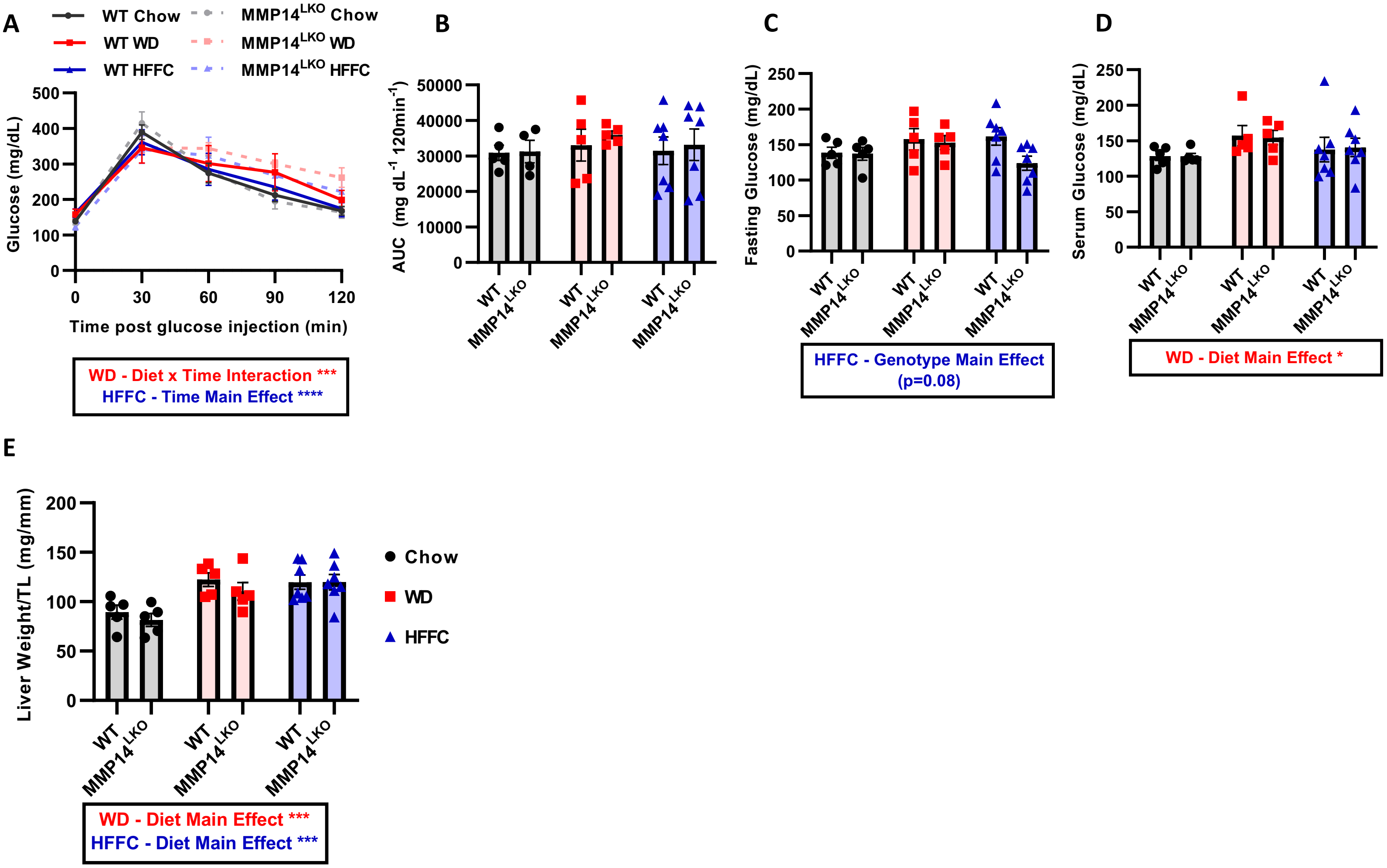

SUPPLEMENTAL FIGURE 4

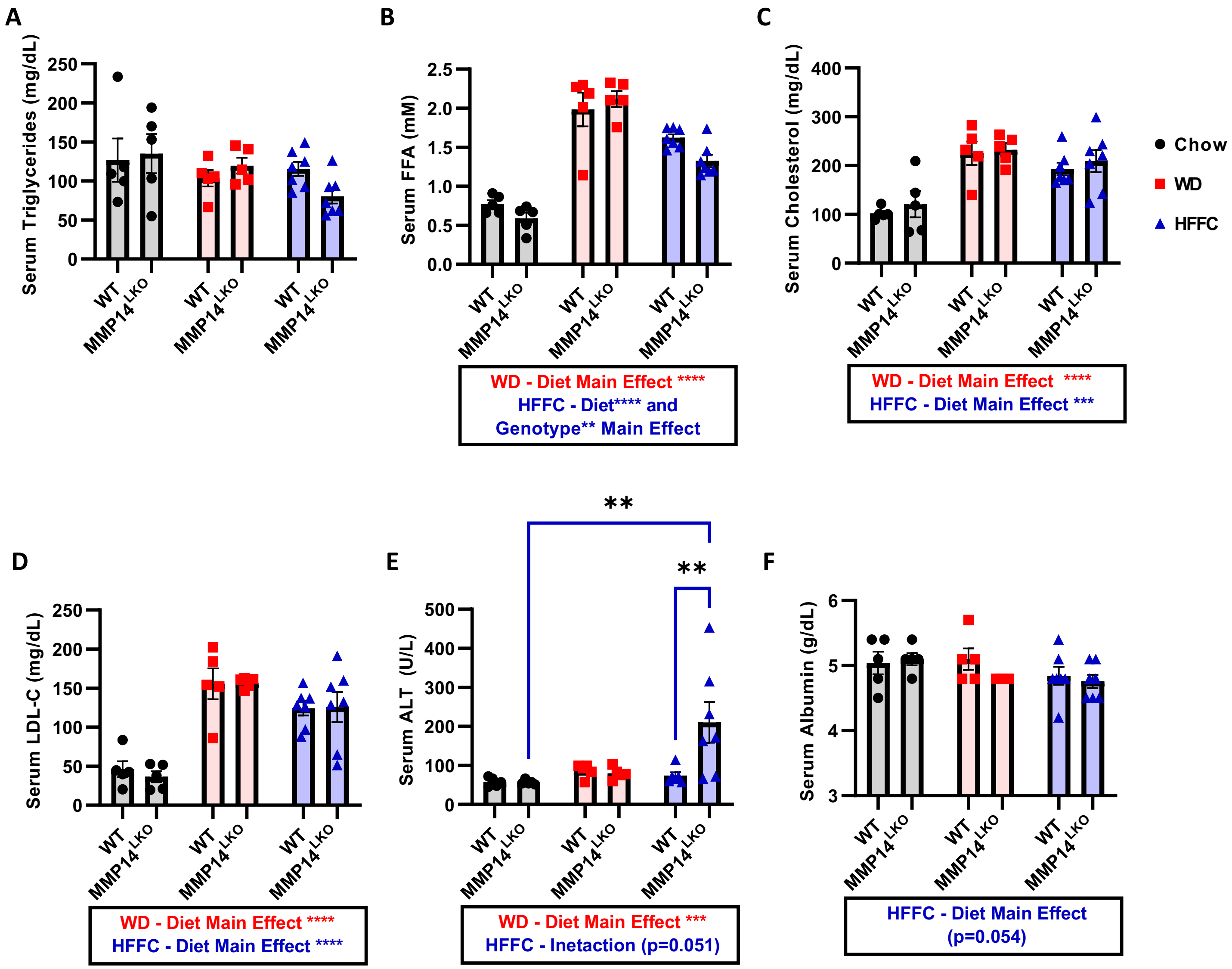

SUPPLEMENTAL FIGURE 5

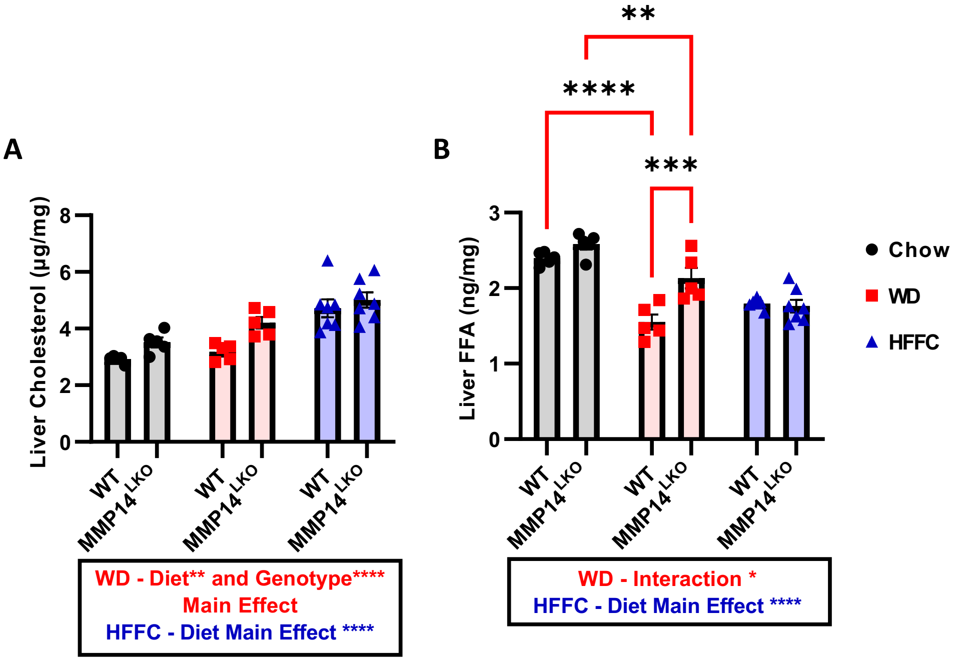

SUPPLEMENTAL FIGURE 6

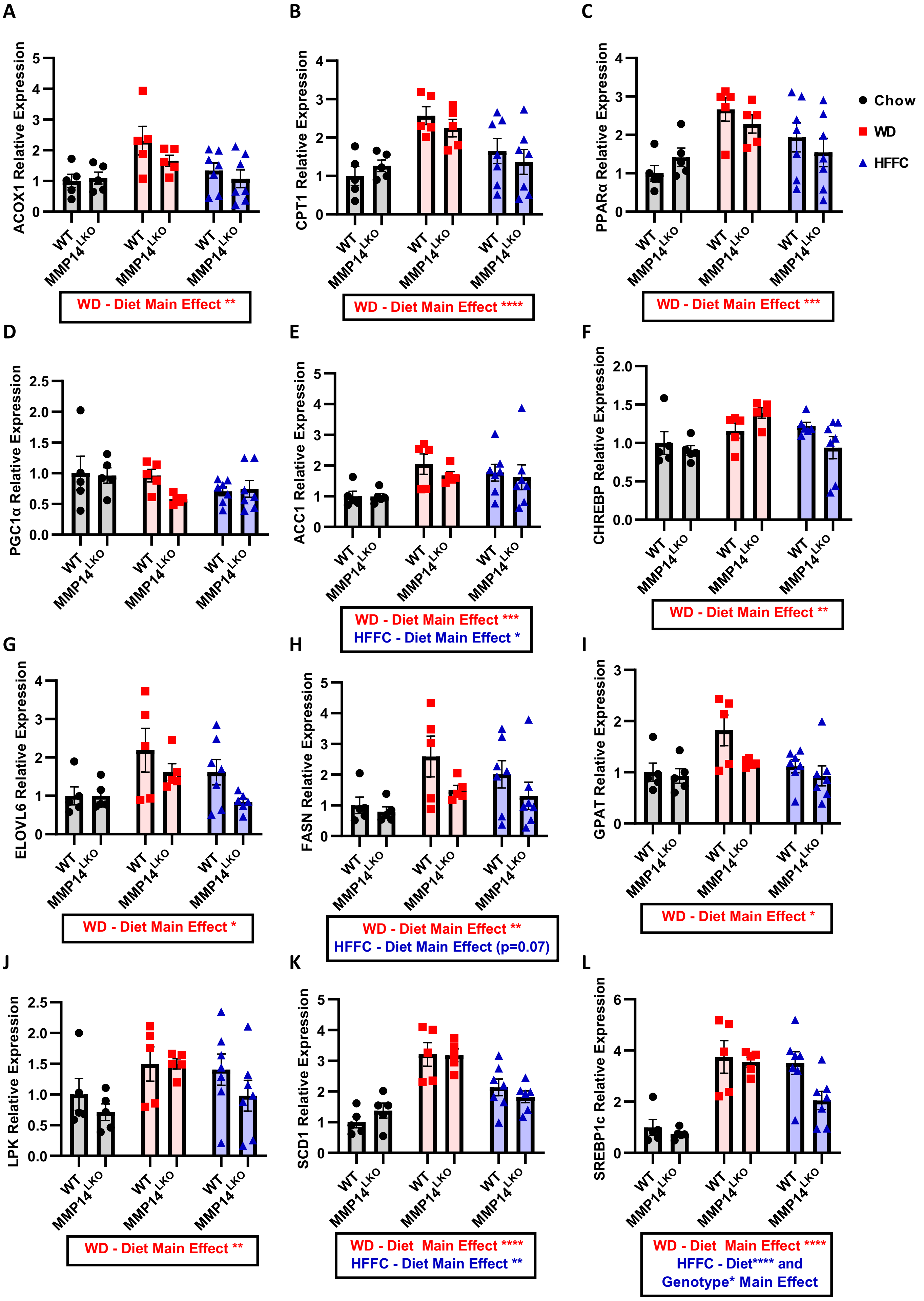

SUPPLEMENTAL FIGURE 7

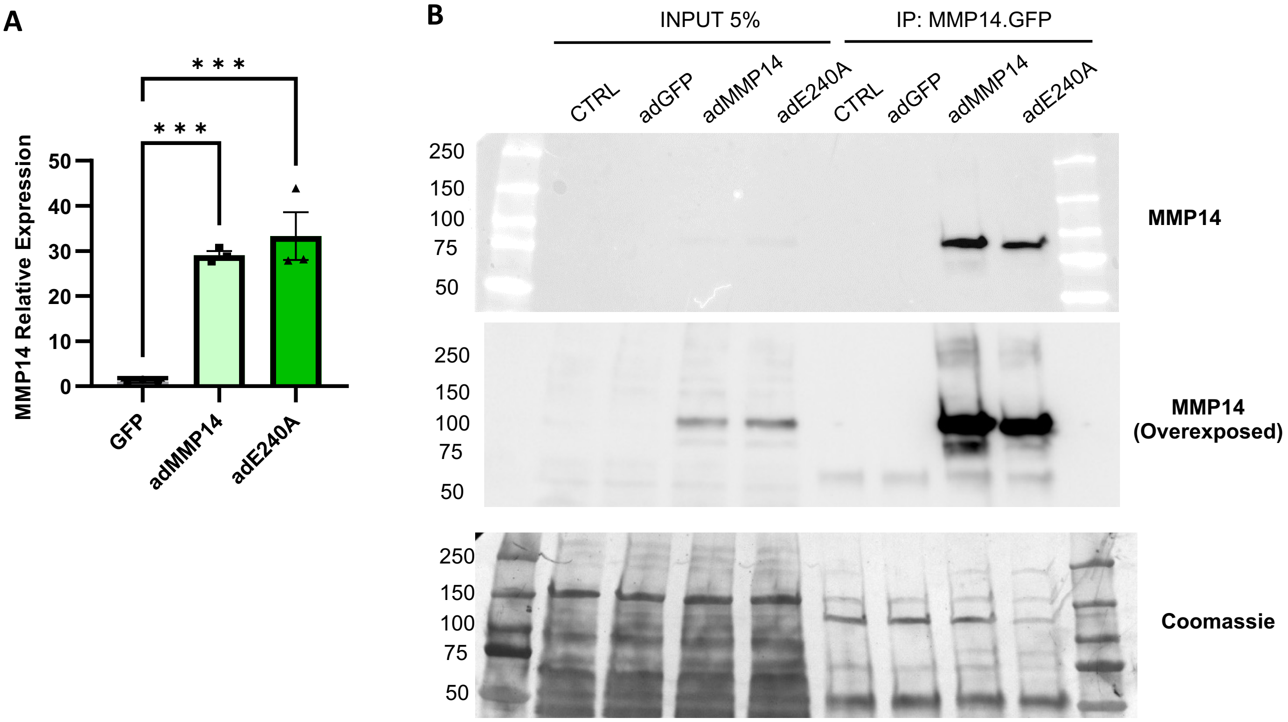

SUPPLEMENTAL FIGURE 8

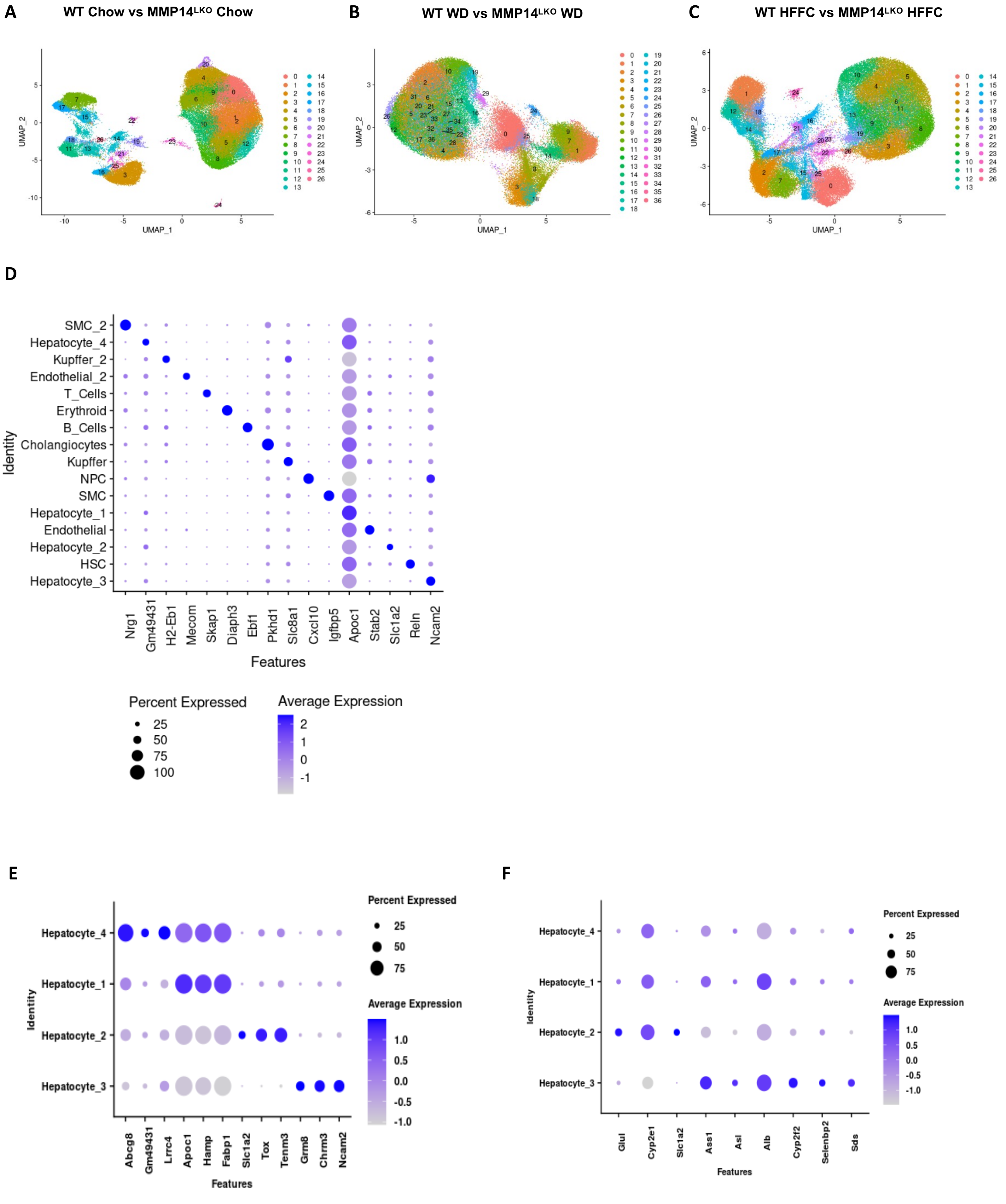

SUPPLEMENTAL FIGURE 9

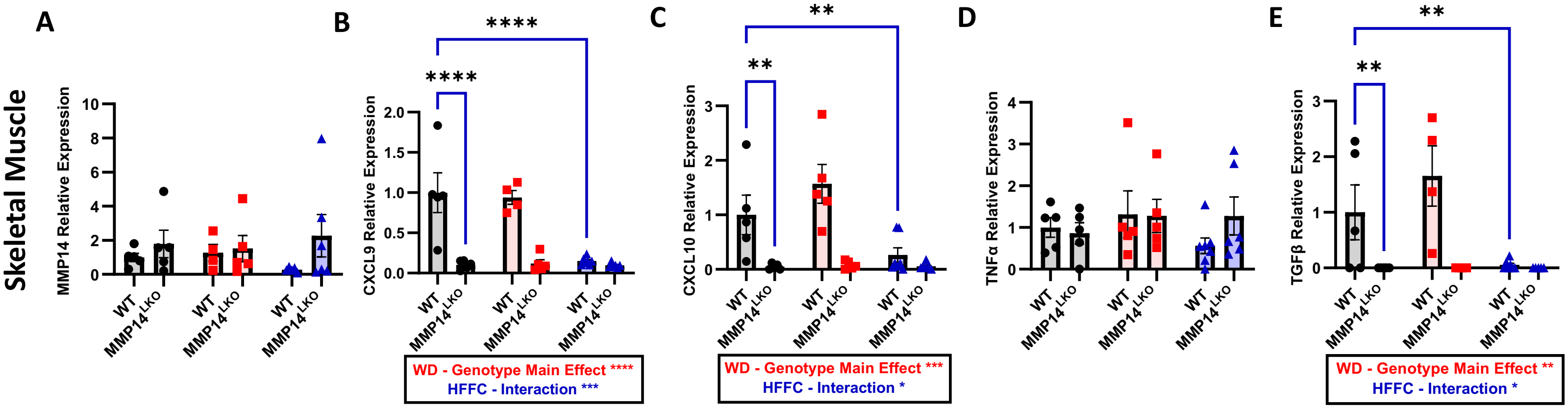

SUPPLEMENTAL TABLE 1. Dietary Components

	CHOW (Lab Diet 5053)	WD (Inotiv TD.88137)	HFFC (Research Diets D09100310)
**Fat**	13.1% kcal	42% kcal	40% kcal
• Andydrous Milkfat	0	21	0
• Palm Oil	0	0	15
• Lard	0	0	2
• Soybean Oil	0	0	3
• Fat (ether extract)	5	0	0
• Fat (acid hydrolysis)	6.3	0	0
**Carbohydrate**	62.4% kcal	42.7% kcal	40% kcal
• Fructose	0	0	22
• Sucrose	2.71	34	11
**Protein**	24.5% kcal	15.2% kcal	20% kcal
**Cholesterol**	135 ppm	0.15	2

Supplemental Table 1. Dietary components and sourcing for Western diet (WD) and high-fat, high-fructose diet (HFFC). Numbers are given as percent by weight unless specified. Diets are iso-caloric. Ppm = parts per million

SUPPLEMENTAL TABLE 2. Primer Sequences

Gene	Forward (5' - 3')	Reverse (5' - 3')
Col1a1	GCT CCT CCT AGG GGC CAC T	CCA CGT CTC ACC ATT GGG G
Col3a1	CTG TAA CAT GGA AAC TGG GGA AA	CCA TAG CTG AAC TGA AAA CAA CC
Col6a3	GCT GCG GAA TCA CTT TGT GC	CAC CTT GAC ACC TTT CTG GGT
Cryl1	GAT TGA CGG CTT CGT CCT GA	ATG ACC AGG TCT AGG TCG CT
CXCL10	CCA AGT GCT GCC GTC ATT TTC	GGC TCG CAG GGA TGA TTT CAA
CXCL2	CCA ACC ACC AGG CTA CAG G	GCG TCA CAC TCA AGC TCT G
LPK	GAA CAT TGC ACG ACT CAA CTT C	CAG TGC GTA TCT CGG GAC C
MMP14	CAG TAT GGC TAC CTA CCT CCA G	GCC TTG CCT GTC ACT TGT AAA
Pde4d	GTG GAG CAT TTG TTG TGT AGT G	CTA GTC CCT AAC TCT TGG CTA TTG
RPL4	CCA AGA CTA TGC GCA GGA ATA	CCT TCT CTG GAA CAA CCT TCT C
Selenbp2	CAA GTG CAA CGT GAG CAA TAC	CTT TGC CAT TAC CCT GGA GAT
TGFβ	CTG CGC TTG CAG AGA TTA AA	GAA AGC CCT GTA TTC CGT CT
Thrsp	CTC CCA ACT TCT CTT CCG TAT C	GAC ATG ACA CCA GGC ACT AA

Supplemental Figures and Table Legends

Fig. S1: Hepatocyte MMP14 does not modulate whole-body energy homeostasis. (**A**) Resting energy expenditure (RER)-Zeitgerber time (ZT) tracing in WT chow (●), $MMP14^{LKO}$ chow (●), WT WD (■), $MMP14^{LKO}$ WD (■), WT HFFC (▲), and $MMP14^{LKO}$ HFFC (▲) during light and dark cycles. (**B-C**) Quantification of RER during the light and dark cycles. (**D**) Heat- Zeitgeber time tracing in WT chow, $MMP14^{LKO}$ chow, WT WD, $MMP14^{LKO}$ WD, WT HFFC, and $MMP14^{LKO}$ HFFC during light and dark cycles. (**E-F**) Quantification of heat during the light and dark cycles. n = 5 for WT chow, $MMP14^{LKO}$ chow, WT WD, and $MMP14^{LKO}$ WD; n = 7 for WT HFFC and $MMP14^{LKO}$ HFFC. Data presented as mean ± SEM. *p < 0.05, **p < 0.01, ***p < 0.001, ****p < 0.0001.

Fig. S2: No change in food intake or calorie intake in chow- and obesigenic diet-fed $MMP14^{LKO}$ mice. (**A-B**) Adipose tissue leptin and adiponectin gene expression in WT and $MMP14^{LKO}$ mice fed chow (●), WD (■), or HFFC (▲). (**C-D**) Mean food intake and calorie intake in WT and $MMP14^{LKO}$ mice fed chow, WD, or HFFC. n = 5 for WT chow, $MMP14^{LKO}$ chow, WT WD, and $MMP14^{LKO}$ WD; n = 7 for WT HFFC and $MMP14^{LKO}$ HFFC. Data presented as mean ± SEM. *p < 0.05, **p < 0.01, ***p < 0.001, ****p < 0.0001.

Fig. S3: Unaltered glucose tolerance in chow- and obesigenic diet-fed $MMP14^{LKO}$ mice. (**A-B**) Glucose tolerance test (GTT) and quantification through area under the curve (AUC) for WT and $MMP14^{LKO}$ mice chow (●), WD (■), or HFFC (▲). (**C**) Glucose levels post 6 h fast prior to GTT. (**D**) Serum glucose levels at time of sacrifice after 17 weeks on diet. (**E**) Liver weights normalized to tibia length (TL). n = 5 for WT chow, $MMP14^{LKO}$ chow, WT WD, and $MMP14^{LKO}$ WD; n = 7 for WT HFFC and $MMP14^{LKO}$ HFFC. Data presented as mean ± SEM. *p < 0.05, **p < 0.01, ***p < 0.001, ****p < 0.0001. Statistical analysis: Two-way ANOVA except: (**A**) three-way ANOVA with repeated measures.

Fig. S4. Serum lipids and biochemistries in chow- and diet-induced obese $MMP14^{LKO}$ mice. Serum triglycerides (**A**), free fatty acids (FFA; **B**), cholesterol (**C**), LDL-C (**D**), alanine aminotransaminase (ALT, **E**), and serum albumin (**F**) for WT and $MMP14^{LKO}$ chow (●), WD (■), and HFFC (▲). n = 5 for WT chow, $MMP14^{LKO}$ chow, WT WD, and $MMP14^{LKO}$ WD; n = 7 for WT HFFC and $MMP14^{LKO}$ HFFC. Data presented as mean ± SEM. *p < 0.05, **p < 0.01, ***p < 0.001, ****p < 0.0001.

Fig. S5. Hepatic cholesterol and non-esterified fatty acid content in chow- and obesigenic diet-fed $MMP14^{LKO}$ mice. Liver cholesterol (**A**) and non-esterified fatty acid (FFA; **B**) per mg of liver tissue for WT and $MMP14^{LKO}$ mice fed chow (●), WD (■), and HFFC (▲). n = 5 for WT chow, $MMP14^{LKO}$ chow, WT WD, and $MMP14^{LKO}$ WD; n = 7 for WT HFFC and $MMP14^{LKO}$ HFFC. Data presented as mean ± SEM. *p < 0.05, **p < 0.01, ***p < 0.001, ****p < 0.0001.

Fig. S6: Hepatocyte MMP14 does not mediate diet-induced changes in hepatic lipid homeostasis gene expression. Shown are qRT-PCR data demonstrating expression of (**A**) ACOX1*,* (**B**) CPT1 (**C**) *PPARA* (**D**) PGC1A, (**E**) ACC1, (**F**) CHREBP, (**G**) ELOVL6, (**H**) FASN, (**I**) GPAT, (**J**) LPK, (**K**) SCD1, and (**L**) SREBP1C in liver tissue for WT and $MMP14^{LKO}$ mice fed chow (●), WD (■), or HFFC (▲). n = 5 for WT chow, $MMP14^{LKO}$ chow,

WT WD, and MMP14^{LKO} WD; n = 7 for WT HFFC and MMP14^{LKO} HFFC. Data presented as mean ± SEM. *p < 0.05, **p < 0.01, ***p < 0.001, ****p < 0.0001.

Fig. S7: Overexpression of MMP14 mRNA and protein after the addition of adMMP14 and adE240A in isolated primary hepatocytes. (**A**) MMP14 mRNA levels in isolated primary hepatocytes treated with adGFP (●), adMMP14 (■), and catalytically inactive MMP14 (adE240A, ▲). (**B**) Immunoprecipitation verification of MMP14 pull-down using adMMP14 and adE240A-tagged-GFP. Coomassie stained membrane was used as loading control. n = 3/group for mRNA. ***p < 0.001, ****p < 0.0001.

Fig. S8: MMP14 shapes the hepatocyte and inflammatory cell population landscape in the steatotic liver. Single-nucleus RNA-seq (snRNA-seq) analysis by UMAP clustering of individual cells sequenced from WT and MMP14^{LKO} mice fed Chow (**A**), WD (**B**), and HFFC (**C**). (**D**) Dot-plot analysis of known cell-specific markers differentiating cell clusters. (**E**) Dot-plot analysis of known cell-specific markers differentiating hepatocyte clusters. (**F**) Dot-plot analysis of markers associated with the pericentral regions (*Glul, Cyp2e1,* and *Slc1a2*) and periportal regions (*Ass1, Asl, Alb, Cyp2f2, Selenbp2*, and *Sds*) of the liver for each hepatocyte subpopulation.

Fig. S9: Loss of hepatic MMP14 attenuates inflammation in skeletal muscle tissue. (**A**) MMP14, (**B**) CXCL9, (**C**) CXCL10, (**D**) TNFα, and (**E**) TGFβ mRNA levels in skeletal muscle for WT and MMP14^{LKO} mice fed chow (●), WD (■), or HFFC (▲). n = 5 for WT chow, MMP14^{LKO} chow, WT WD, and MMP14^{LKO} WD; n = 7 for WT HFFC and MMP14^{LKO} HFFC. Data presented as mean ± SEM. *p < 0.05, **p < 0.01, ***p < 0.001, ****p < 0.0001.

Table S1. Dietary Components. Dietary components and sourcing for Chow, Western diet (WD), and high-fat, high-fructose diet (HFFC). Numbers are given as percent by weight.

Table S2. Primer Sequences.
